# Artemether-Lumefantrine versus Dihydroartemisinin-Piperaquine for Treating Uncomplicated Malaria: A Randomized Trial to Guide Policy in Uganda

**DOI:** 10.1371/journal.pone.0002390

**Published:** 2008-06-11

**Authors:** Adoke Yeka, Grant Dorsey, Moses R. Kamya, Ambrose Talisuna, Myers Lugemwa, John Bosco Rwakimari, Sarah G. Staedke, Philip J. Rosenthal, Fred Wabwire-Mangen, Hasifa Bukirwa

**Affiliations:** 1 Uganda Malaria Surveillance Project, Kampala, Uganda; 2 Department of Medicine, University of California San Francisco, San Francisco, California, United States of America; 3 Makerere University Medical School, Kampala, Uganda; 4 Ministry of Health, Kampala, Uganda; 5 London School of Hygiene and Tropical Medicine, London, United Kingdom; 6 School of Public Health, Makerere University, Kampala, Uganda; London School of Hygiene & Tropical Medicine, United Kingdom

## Abstract

**Background:**

Uganda recently adopted artemether-lumefantrine (AL) as the recommended first-line treatment for uncomplicated malaria. However, AL has several limitations, including a twice-daily dosing regimen, recommendation for administration with fatty food, and a high risk of reinfection soon after therapy in high transmission areas. Dihydroartemisinin-piperaquine (DP) is a new alternative artemisinin-based combination therapy that is dosed once daily and has a long post-treatment prophylactic effect. We compared the efficacy and safety of AL with DP in Kanungu, an area of moderate malaria transmission.

**Methodology/Principal Findings:**

Patients aged 6 months to 10 years with uncomplicated falciparum malaria were randomized to therapy and followed for 42 days. Genotyping was used to distinguish recrudescence from new infection. Of 414 patients enrolled, 408 completed follow-up. Compared to patients treated with artemether-lumefantrine, patients treated with dihydroartemisinin-piperaquine had a significantly lower risk of recurrent parasitaemia (33.2% vs. 12.2%; risk difference = 20.9%, 95% CI 13.0–28.8%) but no statistically significant difference in the risk of treatment failure due to recrudescence (5.8% vs. 2.0%; risk difference = 3.8%, 95% CI −0.2–7.8%). Patients treated with dihydroartemisinin-piperaquine also had a lower risk of developing gametocytaemia after therapy (4.2% vs. 10.6%, p = 0.01). Both drugs were safe and well tolerated.

**Conclusions/Significance:**

DP is highly efficacious, and operationally preferable to AL because of a less intensive dosing schedule and requirements. Dihydroartemisinin-piperaquine should be considered for a role in the antimalarial treatment policy of Uganda.

**Trial Registration:**

Controlled-Trials.com ISRCTN75606663

## Introduction

With the emergence of widespread resistance to chloroquine (CQ) and sulfadoxine-pyrimethamine (SP), most African countries have adopted artemisinin-based combination therapy (ACT) as first-line treatment for uncomplicated malaria. Although several ACTs exist, currently only two have been widely adopted into policy in Africa: artesunate-amodiaquine (AS/AQ) and artemether-lumefantrine (AL), each of which is the recommended therapy for uncomplicated malaria in over a dozen countries [1]. At the time when many countries needed to switch to ACTs, there was limited data comparing ACT efficacy as was the case in Uganda in 2004 when AL was chosen to replace the combination of CQ+SP. Since then several studies in East Africa, including Uganda, have shown AL to be highly efficacious and well-tolerated[2], [3], [4], [5], [6]. AL demonstrated superior efficacy over AS/AQ in all 4 studies that included this comparison[2], [3], [4], [5]. However, AL has several limitations, including a twice-daily dosing regimen, recommendation for administration with fatty food, and a high risk of reinfection soon after therapy in high transmission areas[2].

Dihydroartemisinin-piperaquine (DP) is a fixed-dose ACT which has recently become available in Africa. This drug is relatively inexpensive and has the advantages of once a day dosing and a long post-treatment prophylactic effect[7]. In published studies of over 2,300 patients treated with DP in Asia, this drug was found to be safe and well tolerated, with cure rates consistently exceeding 95%[7].

There have been three published studies of DP in Africa. In all of these studies DP was associated with excellent safety and efficacy, as well as a lower risk of recurrent malaria compared to AS/AQ [8] and AL[9], [10]. One of these studies was done by our group in an area of high transmission in Uganda[9], one in an area of intense seasonal transmission in Burkina Faso[10], and one at 3 sites in Rwanda where the levels of transmission intensity were not specified[8]. Previous data from our group have shown that the efficacy of antimalarial therapy may vary according to the level of transmission intensity, likely due to differences in acquired immunity and the risk of new infections following therapy[11].

In contrast to our previous study done in an area of high transmission intensity, this trial was conducted in an area of moderate transmission intensity in Uganda. We compare the efficacy and safety of DP with the current first-line therapy, AL, testing the hypothesis that the risk of recurrent parasitaemia would differ between the two treatment arms. We also discuss the policy implications of these findings in the context of a growing body of evidence for the ACTs in Africa.

## Methods

The protocol for this trial and supporting CONSORT checklist are available as supporting information; see Checklist S1 and Protocol S1.

### Participants

The study was conducted at Kihihi Health Centre, Kanungu District, Western Uganda. This district experiences perennial mesoendemic malaria; the entomological inoculation rate was measured at 7 infectious bites per person per year[12].

Consecutive patients presenting to the health center with symptoms suggestive of malaria and a positive screening thick blood smear were referred to study physicians for further assessment. Patients were enrolled if they fulfilled the following selection criteria: 1) age 6 months to 10 years; 2) weight ≥5 kg, 3) history of fever in the last 24 hours or axillary temperature ≥37.5°C; 4) no history of serious side effects to study medications; 5) no evidence of a concomitant febrile illness; 6) provision of informed consent by a parent or guardian; 7) no danger signs or evidence of severe malaria; and 8) *P. falciparum* mono-infection with parasite density 2,000–200,000/µl of blood. Because laboratory results were generally not available until the following day, a patient could be excluded after randomization.

### Ethics

The study protocol was approved by the Makerere University Research and Ethics Committee, the Uganda National Council of Science and Technology, and the University of California, San Francisco Committee for Human Research. Parents or guardians of all participating children provided written informed consent before children could be enrolled into the study.

### Interventions

A nurse administered study medications according to weight-based guidelines for fractions of tablets. We administered all drugs orally as follows: AL (Coartem, Novartis, 20 mg artemether/120 mg lumefantrine tablets), administered as one (5–14 kg), two (15–24 kg), three (25–34 kg), or four (≥35 kg) tablets given twice daily for 3 days; DP (Duocotecxin, Holley Pharm, 40 mg dihydroartemisinin/320 mg piperaquine tablets), targeting a total dose of 6.4 and 51.2 mg/kg of dihydroartemisinin and piperaquine, respectively, given in 3 equally divided daily doses to the nearest ¼ tablet (cut with a pill cutter). Participants in the DP group also received placebo tablets administered in the evening over 3 days to simulate the AL dosing schedule. Study medications were administered with water, and patients were given a glass of milk after each dose of study medication. All treatment was directly observed at the study clinic. Participants were given the option either to wait at the clinic for the evening dose (lunch was provided) or to leave and return to the study clinic in the evening (transport was provided). After each dose, children were observed for 30 minutes, and the dose was re-administered if vomiting occurred. All patients were provided with a 3-day supply of acetaminophen for treatment of febrile symptoms. Children with haemoglobin of less than 10 gm/dL were treated according to Integrated Management of Childhood Illness guidelines with ferrous sulfate for 14 days and antihelmintic treatment if appropriate. Households of all patients were given two long-lasting insecticide treated bednets (Permanet, Vestergaarad Frandsen, Denmark) on the day of enrollment, with instructions for one net to be used by the study patient.

Patients were asked to return for follow-up on days 1, 2, 3, 7, 14, 21, 28, 35 and 42, and any other day that they felt ill. Follow-up evaluation consisted of a standardized history and physical examination, including neurological assessment on all days of follow up. We obtained blood by fingerprick for thick blood smears and storage on filter paper on all follow-up days except day 1. Haemoglobin measurement was repeated on day 42 or the day of recurrent symptomatic malaria. If patients did not return for follow-up, they were visited at home.

Treatment failures received quinine (10 mg/kg orally three times a day for 7 days). Patients with evidence of severe malaria or danger signs (convulsions, lethargy, unable to drink or breast feed, repeated vomiting, unable to stand/sit due to weakness) were referred for treatment with parenteral quinine. Patients were excluded during follow-up for use of antimalarial drugs outside of the study, serious adverse events requiring a change in treatment, withdrawal of informed consent, or loss of follow-up (not located within 24 h Days 1–3 or 48 h Days 4–42).

### Laboratory procedures

Initial screening blood smears were stained with 10% Giemsa for 10 minutes. Thick and thin blood smears were stained with 2% Giemsa for 30 minutes. Parasite densities were determined from thick blood smears by counting the number of asexual parasites per 200 white blood cells (WBCs), or per 500 if the count was less than 10 parasites per 200 WBCs, assuming a WBC count of 8,000/µl. A smear was considered negative if no parasites were seen after review of 100 high-power fields. We also assessed for the presence of gametocytes from thick blood smears. Expert microscopists reviewed thin blood smears for non-falciparum infections using known defining characteristics to differentiate between species [13].A second microscopist, who was unaware of the results of the first reading, re-read all slides. A third microscopist unaware of the first two readings resolved discrepant readings. Haemoglobin measurements were made using a portable spectrophotometer (HemoCue, Ängelholm, Sweden).

Molecular genotyping techniques were used to distinguish recrudescent from new infections for all patients with a late clinical failure (LCF) or late parasitological failure (LPF) response. Parasite DNA was isolated from filter paper blood samples collected at enrollment and on the day of recurrent parasitaemia using chelex extraction. Paired samples were genotyped in a stepwise fashion using *msp-2*, *msp-1*, and four microsatellites [14]. A recrudescence was defined as the presence of at least one matched allele at every locus; if at least one locus showed only unmatched alleles, the outcome was classified as a new infection.

### Objectives

The objective of the study was to compare the efficacy and safety of DP with the current first-line therapy, AL, for treating uncomplicated falciparum malaria in an area of moderate transmission intensity in Uganda.

### Outcomes

Treatment outcomes were classified according to 2006 WHO guidelines as early treatment failure (defined as the presence of danger signs, complicated malaria, or failure to adequately respond to therapy on days 0–3), LCF (presence of danger signs, complicated malaria, or fever and parasitaemia on days 4–42), LPF (presence of asymptomatic parasitaemia on days 7–42) or adequate clinical and parasitological response (absence of parasitaemia throughout follow-up[15]. Secondary outcomes included resolution of fever, parasite clearance, change in haemoglobin level, presence of gametocytes during follow-up, and the occurrence of adverse events.

At each follow-up visit study clinicians assessed patients for adverse events and graded them according to scales from the WHO and National Institutes of Health. Adverse events were defined as untoward medical occurrences, following International Conference on Harmonization guidelines, and serious adverse events as experiences resulting in death, life-threatening experience, inpatient hospitalization, persistent or significant incapacity, or medical or surgical intervention to prevent serious outcomes.

### Sample size

We calculated sample size to test the hypothesis that the risk of recurrent parasitaemia after 42 days would differ between the two treatment groups. The risk of recurrent parasitaemia (unadjusted by genotyping) after 42 days was estimated to be 50% after treatment with AL based on previous data[2]. Using this estimate, we calculated that 200 patients (allowing for 10% loss to follow-up) would need to be enrolled in each treatment arm to detect a 15% risk difference between the treatment groups with a two-sided type I error of 0.05 and 80% power.

### Randomization—Sequence generation

A randomization list was computer generated by an off-site investigator. Sequentially numbered, sealed envelopes containing the treatment group assignments were prepared from the randomization list.

### Randomization—Allocation concealment

The study number and assigned treatment were printed on a card and securely sealed in opaque envelopes. Sealed opaque envelopes containing the study number and assigned treatment were secured in a locked cabinet accessible only by the study nurse.

### Randomization—Implementation

The nurse administered treatment by opening an envelope with a matching treatment number sequentially assigned by the study physician.

### Blinding

Only the study nurse was aware of treatment assignments. All other study personnel, including the study physicians and laboratory personnel involved in assessing outcomes, were blinded to the treatment assignments. Patients were not informed of their treatment regimen, but the color and taste of the two study medications were not the same (DP and placebo tablets were light blue; AL tablets were light yellow).

### Statistical methods

Data were entered and verified using Epi-Info version 6.04 and analyzed using STATA version 8.0 (STATA Corporation, College Station, TX, USA). Efficacy and safety data were evaluated using a modified intention-to-treat analysis which included all patients who fulfilled enrollment criteria. Patients who were randomized to therapy but were not enrolled in the study due to laboratory results not being available on day 0 were not included in the analysis. Risks of treatment failure at 28 and 42 days of follow-up (adjusted and unadjusted by genotyping) were estimated using the Kaplan-Meier product limit formula. Data were censored for patients who did not complete follow-up and for new infections when estimating outcomes adjusted by genotyping. Patients with LCF or LPF due to non-falciparum species were censored as non-failures at the time they were classified as LCF or LPF. Comparisons of treatment efficacy were made using risk differences (RD) with exact 95% confidence intervals. Categorical variables were compared using chi-squared or Fisher's exact test and continuous variables were compared using the independent samples t-test. All reported p-values are two sided without adjustment for multiple testing and were considered statistically significant if <0.05.

## Results

### Participant flow

**Figure 1:**
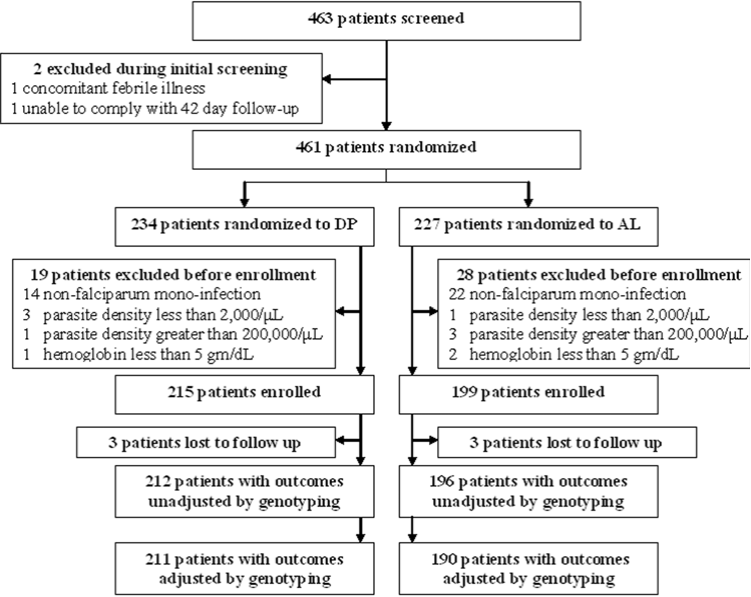
Trial profile comparing antimalarial treatment regimens. AL = artemether-lumefantrine; DP = dihydroartemisinin-piperaquine.

Of 463 patients screened, 2 were excluded during screening and 47 were excluded after treatment assignment, but before enrollment (figure 1). Reasons for exclusion after treatment assignment included non-falciparum malaria infection (n = 36), parasite density <2,000/µl (n = 4) or >200,000/µl (n = 4), and haemoglobin <5.0 g/dL (n = 3). Primary efficacy outcomes, unadjusted and adjusted by genotyping, were available for 408 (98.6%) and 401 (96.9%) enrolled participants, respectively. All the exclusions after enrolment were due to loss to follow-up.

### Recruitment

Study participants were enrolled from August 2006 to April 2007.

### Baseline data

Among patients with treatment outcomes, there were no significant differences in baseline characteristics between the two treatment groups (Table 1).

**Table 1:** Baseline characteristics of patients receiving either dihydroartemisinin-piperaqine (DP) or artemether-lumefantrine (AL)

Characteristic	Treatment group^*^	
	DP (n = 215)	AL (n = 199)
**Female (%)**	**114 (53%)**	**98 (49%)**
**Age in years, median (IQR)**	**2 (0.8–2)**	**2 (1.1–3.5)**
**Age less than 5 years (%)**	**180 (84%)**	**166 (83%)**
**Temperature °C, mean (SD)**	**38.1 (1.3)**	**38.2 (1.3)**
**Parasite density per µL, geometric mean**	**33124**	**35211**
**Haemoglobin gm/dL, mean (SD)**	**9.9 (2.1)**	**9.9 (1.9)**
**Gametocytes present on day 0 (%)**	**12 (5.6%)**	**18 (9.1%)**
**Antimalarial use in previous 2 weeks (%)**	**71 (33%)**	**68 (34%)**

### Numbers analyzed

Efficacy and safety data were evaluated using a modified intention-to-treat analysis, which included all 414 patients who fulfilled enrollment criteria (Table 2).

**Table 2:** WHO treatment outcomes after 42 days of follow-up

Treatment outcomes	Treatment group	
	DP (n = 215)	AL (n = 199)
**Lost to follow-up (no treatment outcome)**	**3 (1.4%)**	**3 (1.5%)**
**Early treatment failure (ETF)**	**0**	**1 (0.5%)**
**Late clinical failure (LCF)**	**9(4.2%)**	**23 (11.6%)**
**Due to new infection with non-falciparum species**	**2**	**5**
**Due to new infection with ** ***P. falciparum***	**6**	**14**
**Due to recrudescence**	**1**	**2**
**Genotyping unsuccessful**	**0**	**2**
**Late parasitological failure (LPF)**	**17 (7.9%)**	**41 (20.6%)**
**Due to new infection with non-falciparum species**	**3**	**14**
**Due to new infection with ** ***P. falciparum***	**10**	**16**
**Due to recrudescence**	**3**	**7**
**Genotyping unsuccessful**	**1**	**4**
**Adequate clinical and parasitological response (ACPR)**	**186 (86.5%)**	**131 (65.8%)**

### Outcomes and estimation

**Figure 2:**
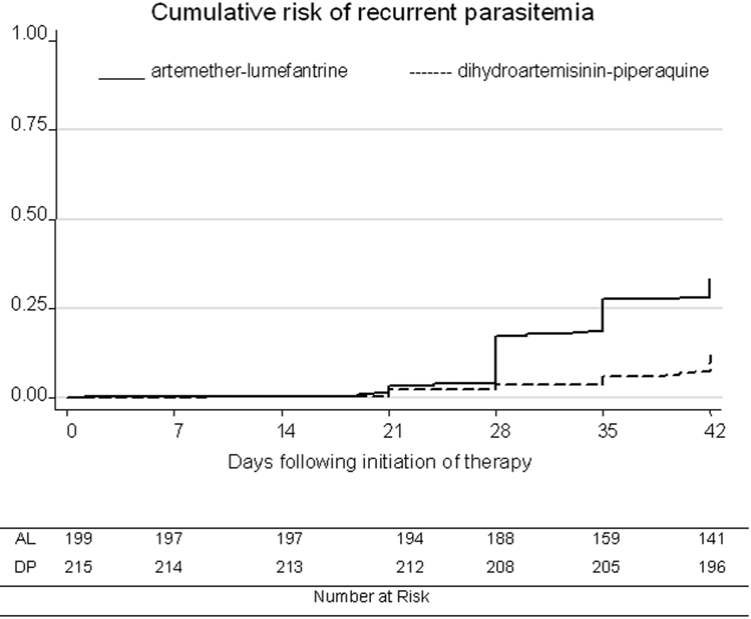
Cumulative risk of recurrent parasitaemia, unadjusted by genotyping. AL = artemether-lumefantrine; DP = dihydroartemisinin-piperaquine.

One participant treated with AL experienced early treatment failure. The child had a febrile convulsion approximately eight hours after administration of the first dose of treatment. Intravenous quinine and supportive management were given and the child recovered completely without any sequelae by day 3. The characteristics of late clinical and parasitological failures are presented in Table 2. Most presumed failures were due to new infections both with *P. falciparum* and non-falciparum species. The risk of treatment failure unadjusted by genotyping was significantly lower for participants treated with DP than for those treated with AL after 28 (3.8% vs. 17.3%; RD = 13.6%, 95% CI 7.7–19.4%) and 42 days of follow up (12.2% vs. 33.2%; RD = 20.9%, 95% CI 13.0–28.8%) (Table 3). Most episodes of recurrent parasitaemia were seen ≥28 days after therapy in the AL group and ≥35 days after therapy in the DP group (Figure 2). After correction by genotyping, there was no statistically significant difference in the risk of treatment failure after 28 days of follow up (0.9% vs. 3.2%; RD = 2.2%, 95% CI −0.6–5.1%); and although the risk of failure tended to be lower in those treated with DP than for AL after 42 days of follow up, the difference did not reach statistical significance(2.0% vs. 5.8%; RD = 3.8%, 95% CI −0.2–7.8%), (Table 3).

**Table 3:** Estimates of comparative efficacy

Risk category	DP (95% CI)	AL (95% CI)	RD (95% CI)	p-value
28d risk of treatment failure unadjusted by genotyping*	3.8% (1.9–7.4%)	17.3% (12.7–23.4%)	13.6% (7.7–19.4%)	<0.0001
28d risk of treatment failure adjusted by genotyping†	0.9% (0.2–3.7%)	3.2% (1.4–7.0%)	2.2% (−0.6–5.1%)	0.12
42d risk of treatment failure unadjusted by genotyping*	12.2% (8.5–17.5%)	33.2% (27.0–40.2%)	20.9% (13.0–28.8%)	<0.0001
42d risk of treatment failure adjusted by genotyping†	2.0% (0.7–5.1%)	5.8% (3.1–10.5%)	3.8% (−0.2–7.8%)	0.06

* Any ETF, LCF or LPF

† Any ETF and LCF or LPF due to recrudescence

The proportion of patients with fever on Day 1 was significantly lower in participants treated with DP (55% vs 68%, P = 0.01), but was similar over the second and third days of follow up in the two treatment groups. Both treatments produced rapid clearance of parasitaemia, with no parasites detected by day 3 (Table 4). The appearance of gametocytes not present at enrollment was significantly lower over the last 4 weeks of follow-up in participants treated with DP (Table 4). This can be explained by differences in the risk of recurrent parasitaemia as the risk of developing gametocytes after therapy was significantly higher in patients with recurrent parasitaemia compared to those without recurrent parasitaemia in both the AL (34% vs. 1%, p<0.0001) and DP (24% vs. 2%, p<0.0001) treatment arms.

**Table 4:** Secondary outcomes

Category	Outcome	Treatment group		
		DP (n = 215)	AL (n = 199)	p-value
Fever clearance*	Fever on day 1	117/213 (55%)	133/197 (68%)	0.01
	Fever on day 2	44/213 (21%)	37/197 (19%)	0.71
	Fever on day 3	22/213 (10%)	22/197 (11%)	0.87
Parasite clearance	Parasitaemia on day 2	7/213 (3.3%)	5/197 (2.5%)	0.77
	Parasitaemia on day 3	0	0	-
Appearance of gametocytes not present on day 0	Days 1–14.	4/201 (2.0%)	1/179 (0.6%)	0.38
	Days 15–28	1/200 (0.5%)	7/178 (3.9%)	0.03
	Days 29–42	4/194 (2.1%)	13/147 (8.8%)	0.005
Haemoglobin (Hb) recovery†	Mean increase (SD) in Hb (g/dL)	1.75 (1.8)	1.66 (2.0)	0.63
Patients with adverse events of any severity	Cough	164/213 (77%)	150/198 (76%)	0.77
	Coryza	159/213 (75%)	150/198 (76%)	0.80
	Abdominal pain	17/74 (23%)	24/63 (38%)	0.05
	Anorexia	47/213 (22%)	49/198 (25%)	0.52
	Vomiting	35/213 (16%)	35/198 (18%)	0.74
	Weakness/malaise	28/213 (13%)	27/198 (14%)	0.88
	Diarrhea	26/213 (12%)	23/198 (12%)	0.85
	Pruritis	8/213 (4%)	3/198 (1.5%)	0.16
Patients with serious adverse events		5/215 (2.3%)	2/199 (1.0%)	0.45

* Subjective fever over previous 24 hours or temperature ≥37.5°C

† Change in Hb from day 0 to day 42 or day of clinical failure

### Adverse events

No patients were withdrawn from the trial for vomiting that would have required alternative treatment. Adverse events, broadly defined as any untoward medical occurrences, occurred commonly. Most adverse events were of mild or moderate severity and consistent with symptoms due to malaria. The most commonly reported adverse events in both treatment groups were cough, coryza, abdominal pain, anorexia, weakness, diarrhea and pruritus (Table 4). Adverse events were not significantly different between the two treatment groups. A total of 7 serious adverse events were reported in 7 patients. The distribution of the serious adverse events was not significantly different in the two treatment groups, except that an increased incidence of abdominal pain with AL was of borderline significance. Serious adverse events included convulsions (n = 2), pyomyositis (n = 2), vomiting (n = 1), severe anaemia (n = 1), and dehydration secondary to vomiting (n = 1). All serous adverse events were judged to be unrelated to study medications. No deaths occurred in this study.

## Discussion

### Interpretation

In this study, we compared locally relevant antimalarial drug combinations in a randomized trial of children with uncomplicated malaria, and followed up patients for 42 days. AL is currently the first line treatment for malaria in Uganda

DP is a newer fixed combination regimen that is registered in Uganda. Both regimens were highly efficacious in clearing initial *P. falciparum* infections in children. However patients treated with DP had a significantly reduced risk of treatment failure due to recurrent parasitaemia and a lower risk of recurrent parasitaemia due to recrudescence.

The risk of treatment failure unadjusted by genotyping, which was significantly different between the two treatment arms, largely reflects a difference in rates of reinfection, rather than recrudescence. DP clearly offered a better post treatment prophylactic effect following therapy compared to AL. The significant lower risk of recurrent parasitaemia after treatment with DP is likely explained by differences in pharmacokinetics of the non-artemisinin partner drugs. Piperaquine, a bisquinoline, is estimated to have an elimination half-life of 2–3 weeks [16]; lumefantrine, an aryl alcohol, has an estimated elimination half-life of 4–10 days [17]. The performance observed for DP is consistent with results from other recent studies in Africa [8], [9], [10] and prior studies in Asia [18], [19], [20], [21], suggesting that this combination may be highly effective in areas with considerable resistance to other antimalarial drugs. Other benefits offered by DP compared to AL include simpler dosing, more consistent intestinal absorption, relatively low cost, a lower risk of gametocytaemia after therapy and better haemoglobin recovery [7]. Our study also offered a rigorous comparison of the safety and tolerability of the two tested regimens. Although, when defined as any untoward medical occurrences, adverse events were common, drug-related adverse events appeared to be uncommon and generally mild.

Five years after the call for deployment of artemisinin combination therapy for treating malaria first gained momentum [22], the strategic use of ACTs is now broadly accepted. A remaining challenge, however, is the choice of ACT for first line therapy in a particular country [23]. The WHO currently recommends four ACTs for uncomplicated malaria; AS+mefloquine, which is impractical for Africa due to high cost and risk of toxicity; AS+sulfadoxine/pyrimethamine (SP), which showed unacceptably poor efficacy in Uganda [24], probably due to frequent parasite resistance to SP; AS+AQ, and AL. In addition, DP is a newer ACT with excellent efficacy. In studies in Uganda, AS+AQ was inferior to AL, with increased recrudescence in Kampala [3] and increased recurrent (mostly new) infections over 28 days in Tororo, a site with very high transmission [2]. AS+AQ thus appears to be inferior to AL for treating uncomplicated malaria in Uganda, probably due to limited post-treatment prophylaxis with this regimen and significant resistance of malaria parasites to AQ. However AL has important limitations, including the need for twice-daily dosing, irregular pharmacokinetics, and high rates of new infections over 28–42 days in high transmission areas [2], [9], and consideration of DP as a superior therapy for uncomplicated malaria is warranted.

### Generalizability

This study was conducted in a moderate transmission area, but the results are consistent with those from studies from high transmission sites in Africa [8], [9], [10]. Therefore considered together, the evidence adduced can be applied to other malaria settings in Africa.

### Overall evidence

Our results add to recent data comparing DP to other artemisinin combination treatments of interest. In this study, DP is clearly superior to AL at preventing new malaria infections; is at least as safe as AL; and with simpler dosing requirements and lower cost, it appears to be a preferable alternative.

This study adds data from a moderate transmission site to two prior studies at high transmission sites in Africa [8], [9], [10] including one very high transmission site in Uganda [9], all showing superior efficacy for DP over AL, due to a lower risk of recurrent malaria after therapy. This raises the question of what role DP should play in Uganda's antimalarial treatment policy. Although the question of whether ACTs can be safely and effectively introduced into the home based management of fever programme (HBMF) has not been fully answered, and recognizing the challenges of changing drug policy, it is possible that DP could be introduced into HBMF instead of AL as planned. Additionally DP could replace AQ +AS as the official first line alternative in Uganda, and possibly other countries in Africa.

## Supporting Information

Protocol S1 Trial Protocol
(1.07 MB DOC)

Checklist S1 CONSORT Checklist
(0.06 MB DOC)
