# Supplementary material for: Artemether-Lumefantrine versus Dihydroartemisinin-Piperaquine for Treating Uncomplicated Malaria: A Randomized Trial to Guide Policy in Uganda
Source: PLoS One. 2008 Jun 11;3(6):e2390. doi: 10.1371/journal.pone.0002390 (PMC2405936; doi:10.1371/journal.pone.0002390)
Supplement: Protocol S1 — Trial Protocol (1.07 MB DOC) [file pone.0002390.s001.doc]

**COMPARISON OF ARTEMETHER-LUMEFANTRINE AND DIHYDROARTEMISININ-PIPERAQUINE FOR TREATMENT OF UNCOMPLICATED MALARIA IN UGANDA:**

**EVALUATION OF EFFICACY, SAFETY, AND TOLERABILITY AT THREE SITES WITH VARYING TRANSMISSION INTENSITY**

**Protocol version 1.1**

**Uganda Malaria Surveillance Project Drug Efficacy Surveillance Studies**

**December 11^th^, 2005**

TABLE OF CONTENTS

ABBREVIATIONS and ACRONYMS 5

STUDY PERSONNEL 6

1.0 STUDY SUMMARY 8

2.0 INTRODUCTION 9

2.1 Background 9

2.1.1 Burden of malaria in Uganda 9

2.1.2 Antimalarial drug efficacy surveillance studies in Uganda 9

2.1.3 Uganda Malaria Surveillance Project (UMSP) drug efficacy surveillance studies 10

2.1.4 UMSP infrastructure 10

2.1.5 Characteristics of sentinel sites and patient populations 11

2.1.6 Results of UMSP antimalarial drug efficacy studies 12

2.1.7 Current antimalarial drug policy in Uganda 14

2.2 Rationale 15

2.3 Study drugs 15

2.3.1 Artemether-lumefantrine (AL) 15

2.3.2 Dihydroartemisinin-piperaquine (DP) 15

3.0 STUDY AIMS 16

4.0 STUDY DESIGN 16

4.1 Overall study design 16

4.2 Study outcome and assessment 16

4.2.1 Classification of treatment outcome 16

4.2.2 Primary outcomes 16

4.2.3 Secondary outcomes 16

4.3 Randomization 17

5.0 PARTICIPANT SELECTION AND ENROLLMENT 17

5.1 Study sites 17

5.2 Malaria surveillance, screening and recruitment process 17

5.3 Selection criteria 18

6.0 BASELINE EVALUATION AND TREATMENT ALLOCATION 18

6.1 Baseline evaluation and procedures 18

6.2 Treatment group assignment 19

6.3 Treatment allocation 19

6.4 Study drugs 19

6.4.1 Dosing of study drugs 19

6.4.2 Blinding 20

6.4.3 Administration of study drugs 20

6.5 Additional medicaitons 20

7.0 FOLLOW-UP EVALUATION AND PROCEDURES 20

7.1 Follow-up schedule 20

7.2 Classification of treatment outcome and management of treatmnt failure 21

7.3 Exclusion and enrollment and loss to follow-up 21

7.4 Management of non-malarial illnesses 21

7.5 Distribution of insecticide treated bednets (ITNs) 22

8.0 ASSESSMENT OF ADVERSE EVENTS 22

8.1 Definitions 22

8.2 Identification of adverse events 22

8.3 Reporting of adverse events 22

8.4 Reporting of serious adverse events 23

9.0 STATISTICAL CONSIDERATIONS 23

9.1 Sample size calculations 23

9.2 Analytical plan 23

10.0 DATA COLLLECTION AND MANAGEMENT 23

10.1 Data management 23

10.2 Data quality assurance and monitoring 24

10.3 Records 24

11.0 LABORATORY PROCEDURES 24

11.1 Blood smears 24

11.2 Hemoglobin measurements 24

11.3 Molecular studies 24

12.0 PROTECTION OF HUMAN SUBJECTS 25

12.1 Institutional Revies Board (IRB) review and informed consent 25

12.2 Risks and discomforts 25

12.2.1 Privacy 25

12.2.2 Risks of randomization 25

12.2.3 Fingerprick blood draws 25

12.2.4 Risk of artemisinins 25

12.2.5 Risk of artemether-lumefantrine (AL) 26

12.2.6 Risk of dihyroartemisinin-piperaquine (DP) 27

12.2.7 Risk of quinine 27

12.3 Compensation 27

12.4 Consent procedures 28

12.5 Alternatives 28

12.6 Confidentiality of records 28

13.0 CLINICAL AND SAFETY MONITORING 28

13.1 Date and safety monitoring board 28

13.2 Monitoring plan 28

13.3 Stopping guidelines 28

13.4 Reporting of serious adverse events from sentinel sites to Kampala 28

13.5 Management of serious adverse events 29

14.0 TIMETABLE 29

15.0 REFERENCES 29

Appendix 1 33

Appendix 2 34

Appendix 3 35

Appendix 4 36

Appendix 5 37

Appendix 6a 45

Appendix 6b 46

Appendix 7 47

Appendix 8 48

Appendix 9 49

Appendix 10 53

Appendix 11 54

Appendix 12 55

Appendix 13 59

# ABBREVIATIONS and ACRONYMS

UMSP: Uganda Malaria Surveillance Project

MOH: Ministry of Health

EANMAT: East African Network for Monitoring Antimalarial Therapy

AL: Artemether-lumefantrine

DP: Dihydroartemisinin-piperaquine

US: United States

UCSF: University of California San Francisco

WHO: World Health Organization

ACPR: Adequate clinical and parasitological response

ETF: Early treatment failure

LCF: Late clinical failure

LPF: Late parasitological failure

IMCI: Integrated Management of Childhood Illness

Hb: Hemoglobin

IRB: Institutional Review Board

DNA: Deoxyribonucleic acid

GCP: Good Clinical Practice.

STUDY PERSONEL

| Name | Institution | Title |
| --- | --- | --- |
| Fred Wabwire-Mangen, MBChB, MPH, PhD | MU | Project Director |
| Moses Kamya, MBChB, MMed, MPH | MU | Co-Project Director |
| Fred Kironde, MSc, PhD | MU | Technical Advisor |
| Samuel Nsobya, MSc | MU | Chief Laboratory Technologist |
| John B. Rwakimare, MBChB, MPH | Uganda MOH | Technical Advisor |
| Ambrose Talisuna, MBChB, MSc, PhD | Uganda MOH | Technical Advisor |
| Thomas Kator, MBChB, MPH | Uganda MOH | Technical Advisor |
| Patrobas Mufubenga, MBChB, MPH | Uganda MOH | Technical Advisor |
| Adoke Yeka, MBChB, MPH | UMSP | Epidemiologist |
| Hasifa Bukirwa, MBChB, MPH | UMSP | Epidemiologist |
| John Patrick Mpindi | UMSP | Data Manager |
| Nelson Budaka | UMSP | Laboratory Technologist |
| Catherine Tugaineyo | UMSP | Administrator |
| Nuhu Kibampawo | UMSP | Driver |
| TBH (2) | UMSP | Medical Officer |
| TBH (2) | UMSP | Nurses |
| TBH (2) | UMSP | Laboratory Technologist |
| TBH (2) | UMSP | Home Visitor |
| Grant Dorsey, MD, PhD | UCSF | U.S. Principal Investigator |
| Philip Rosenthal, MD | UCSF | U.S. Co-principal Investigator |
| Sarah Staeke, MD, DTM&H | UCSF | U.S. Co-principal Investigator |
| Heidi Hopkins, MD, MPH | UCSF | Technical Advisor |
| Purba Chatterjee, MPH | UCSF | U.S. Administrator |

**Names and contact information of investigators**

Name: Fred Wabwire Mangen, MBChB, DTM&H, MPH, PhD

Institution: Makerere University Institute of Public Health

Address: Institute of Public Health, P.O. Box 7072, Kampala, Uganda

Phone Number: 256-41-543872

Fax Number: 256-41-531807

Email: fwabwire@iph.ac.ug

Project role: Ugandan Principal Investigator.

Name: Moses Kamya, MBChB, M Med, MPH

Institution: Makerere University Medical School

Address: Department of Medicine, P.O. Box 7072, Kampala, Uganda

Phone Number: 256-41-541188

Fax Number: 256-41-540524

Email: [malaria@infocom.co.ug](mailto:malaria@infocom.co.ug)

Project role: Ugandan Co–Principal Investigator.

Name: Fred Kironde, PhD

Institution: Makerere University Medical School

Address: Department of Biochemistry Medical Biochemistry Bldg. P.O. Box 7072, Kampala, Uganda

Phone Number: 256-41-530555

Fax Number: 256-41-534314

Project role: Collaborating Ugandan Investigator, Director of MU-UCSF laboratory activities

Name: Grant Dorsey, MD, MPH, PhD

Institution: University of California, San Francisco

Address: UCSF, Box 0811, San Francisco, CA 94143

Phone Number: 415-206-8687

Fax Number: 415-648-8425

Email: [grantd@itsa.ucsf.edu](mailto:staedke@itsa.ucsf.edu)

Project role: Collaborating US Principal Investigator.

Name: Sarah G. Staedke, MD, DTM&H

Institution: University of California, San Francisco

Address: MU-UCSF, P.O. Box 7475, Kampala, Uganda

Phone Number: 256-77-604844

Fax Number: 256-41-540524

Email: [staedke@itsa.ucsf.edu](mailto:staedke@itsa.ucsf.edu)

Project role: Co- Investigator

Name: Philip Rosenthal, MD

Institution: University of California, San Francisco

Address: UCSF, Box 0811, San Francisco, CA 94143

Phone Number: 415-206-8845

Fax Number: 415-648-8425

Email: [rosnthl@itsa.ucsf.edu](mailto:rosnthl@itsa.ucsf.edu)

Project role: Co – Investigator.

# 1.0 STUDY SUMMARY

| **Title** | **Comparison of Artemether-lumefantrine and Dihydroartemisinin-piperaquine for treatment of uncomplicated malaria in Uganda: evaluation of efficacy, safety, and tolerability** |
| --- | --- |
| **Description** | Randomized, single-blinded trials of two leading new antimalarial regimens at three sites with varying transmission intensity |
| **Participants and sample size** | Ugandans aged > 6 months – 10 years  Total sample size 1200 subjects, 400 per site  (2 treatment arms at each site with 200 subjects per treatment arm) |
| **Clinical Sites** | The study will be conducted at 3 Uganda Malaria Surveillance Project (UMSP) sentinel sites namely Apac, Kanungu and Mubende. |
| **Selection Criteria** | 1. Not previously enrolled in this study 2. Age > 6 months – 10 years 3. Weight > 5 kg 4. Fever (> 37.5ºC axillary) or history of fever in the previous 24 hours 5. Absence of any history of serious side effects to study medications 6. No evidence of a concomitant febrile illness 7. Provision of informed consent and agreement to follow-up for 42 days 8. No evidence of severe malaria or danger signs 9. Absence of repeated vomiting of study medications on day 0 10. *P. falciparum* mono-infection 11. Parasite density > 2000/ul and < 200,000/ul |
| **Study intervention** | Subjects will be randomized to treatment with Artemether-lumefantrine (AL) or Dihydroartemisinin-piperaquine (DP). Subjects in the DP arm will also receive placebo tablets to ensure that the number of doses received is identical in the two treatment groups. Subjects who fail initial therapy will receive quinine, the standard treatment for recurrent malaria in Uganda. |
| **Follow-up** | Subjects will be followed for 42 days and will be asked to return for follow-up assessment on days 1, 2, 3, 7, 14, 21, 28, 35, 42 and any unscheduled day that they feel ill. |
| **Primary outcomes** | Risk of treatment failure unadjusted and adjusted by genotyping at day 42 |
| **Analytical plan** | Primary outcomes will be based on a modified intention-to-treat analysis. Outcomes will be classified according to 2003 WHO criteria as early treatment failure (ETF), late clinical failure (LCF), late parasitological failure (LPF), and adequate clinical and parasitological response (ACPR). Treatment failure unadjusted by genotyping will be defined as any ETF, LCF, or LPF. Treatment failure adjusted by genotyping will be defined as any ETF and any LCF or LPF due to recrudescence. Risks of treatment failure will be estimated using the Kaplan-Meier product limit formula with censoring for patients who do not complete the study or have an outcome of interest. |
| **Secondary outcomes** | 1. Prevalence of fever on days 1-3 2. Prevalence of parasitemia on days 2 and 3 3. Change in mean hemoglobin level between days 0 and 42 (or day of treatment failure) 4. Prevalence of gametocytes during follow-up 5. Risk of serious adverse events during follow-up 6. Risk of adverse events of moderate or greater severity, at least possibly related to the study medications, excluding patients requiring quinine therapy 7. Selection of molecular markers associated with drug resistance |

# 2.0 INTRODUCTION

**2.1 Background**

**2.1.1 Burden of malaria in Uganda**

Malaria is endemic in 95% of Uganda. The remaining 5% are epidemic-prone areas in the highlands of the Southwest and East. Malaria is the leading cause of morbidity and mortality in the country, accounting for 25-40% of all outpatient visits at health facilities, 20% of hospital admissions, and 9-14% of inpatient deaths (Uganda MOH, unpublished). A recent MOH information update on malaria in Uganda (2000) reported that malaria morbidity is increasing (25-40% of outpatient visits in 1992-3, 27-51% in 1998 and 29-50% in 1999.). Children under five and pregnant women bear the greatest burden of the disease and within these groups the poorest are most vulnerable. A 1995 Burden of Disease report indicated that 15.4% of life years lost to premature death were due to malaria. Malaria treatment in poor families consumes a large proportion of limited household budgets. Malaria stricken families spend 25% of their income on direct or indirect costs of the disease*.* Time off work and transport to seek treatment further reduces household income. Poor school performance due to malaria illness and absenteeism reduces children’s chances of escaping from poverty. Poor people tend to live in environments conducive to mosquito breeding and malaria transmission. Thus malaria enhances poverty, which in turn causes poor disease management, locking people in a malaria-poverty trap.

**2.1.2 Antimalarial drug efficacy surveillance studies in Uganda**

Chloroquine (CQ) has been the standard antimalarial in Africa for many years, but resistance to this agent is now very common. In the 1990s a few African countries changed their recommendations for primary therapy for uncomplicated malaria from CQ to sulfadoxine-pyrimethamine (SP).^1^ However, resistance to SP has spread rapidly, especially in East Africa.^2,3^ Prior reports have suggested that, compared to the rest of East Africa, antimalarial drug resistance in Uganda had been relatively uncommon, presumably due to the isolation of the country during the 1970s and 1980s and limited drug use due to political instability. Two studies done between 1988 and 1993 in children from 9 different sites reported the prevalence of resistance to range from 3-39% for CQ and 0-5% for SP.^4,5^ However, these studies had major limitations, in that they were largely limited to asymptomatic infections and likely under-stated resistance levels due to short follow-up periods (7 days). Two studies done in rural and urban areas in 1996 using symptomatic patients and more standardized methodology (albeit with 14-day follow-up that is now deemed too short to recognize all treatment failures) documented 36-58% resistance to CQ and 6-13% resistance to SP.^6,7^ In response to the relative paucity of antimalarial drug efficacy data and concern that resistance was increasing, the Malaria Control Division of the Ugandan MOH initiated studies of CQ and SP resistance at 7 sentinel sites around the country. These sites were selected in collaboration with the East African Network for Monitoring Antimalarial Treatment (EANMAT) based on endemicity, geographical representation, ease of supervision, and availability of suitable staff. Protocols were standardized to study symptomatic children under the age of five with clinical failure as the primary outcome. There were nonetheless several limitations to these early studies, including a 14-day follow-up period, which likely underestimated the true risk of treatment failure, small sample sizes, lack of fully comparative study designs, and the absence of molecular genotyping data to distinguish recrudescence (true treatment failure) from new infections. Results of these studies, conducted from 1998-1999, indicated high levels of resistance to chloroquine (9-44%) and moderate levels of resistance to SP (0-18%). Based on the results of these studies and others, in 2000 the Uganda MOH chose to switch from CQ to a combination of CQ + SP as the recommended first-line therapy for uncomplicated malaria.^8^ Of note, at the time this decision was made there were no studies on the efficacy of CQ + SP in Uganda, reflecting major limitations in data for evidence-based decision making.

**2.1.3 Uganda Malaria Surveillance Project (UMSP) drug efficacy surveillance studies.** UMSP was established in 2001 with the purpose of creating a multi-site surveillance system in Uganda for evaluating the efficacy of available antimalarial therapies and monitoring adverse events secondary to therapy. In addition, this program was designed to enhance local capacity and expand existing infrastructure with the goal of providing sustainable progress in malaria control. In recognition of the need for high quality drug efficacy studies, UMSP carried out studies at the 7 previously established sentinel sites (Figure 1). These studies were designed to collect “state of the art” drug efficacy surveillance data with large sample sizes, 28-day follow-up, molecular genotyping to distinguish recrudescence from new infections, systematic collection of data on drug safety and tolerability, quality control, and standardized outcomes based on WHO criteria. The studies were carried out in collaboration with the Uganda Malaria Control Program to assist in the development of evidence-based antimalarial treatment policies.

**2.1.4 UMSP infrastructure.** A core facility was established in Kampala, Uganda including administrative offices, a designated malaria laboratory, and a data management center. The core facility supports weekly meetings of principal investigators, project coordination, and training activities. Study teams are organized to travel to the sentinel sites to carry out the surveillance studies as described below. These teams included study physicians, laboratory technologists, nurses, and home visitors. At the sentinel sites study teams work with existing staff at the district health centers to build capacity and create a sustainable program. Study coordinators from the core facilities make frequent visits to the field sites to deliver supplies, provide on site training, and gather case record forms and laboratory samples for data quality control and management in Kampala. This system has created a highly experienced team of Ugandans and the infrastructure necessary for continued surveillance.

**Figure 1. UMSP sentinel sites**

**
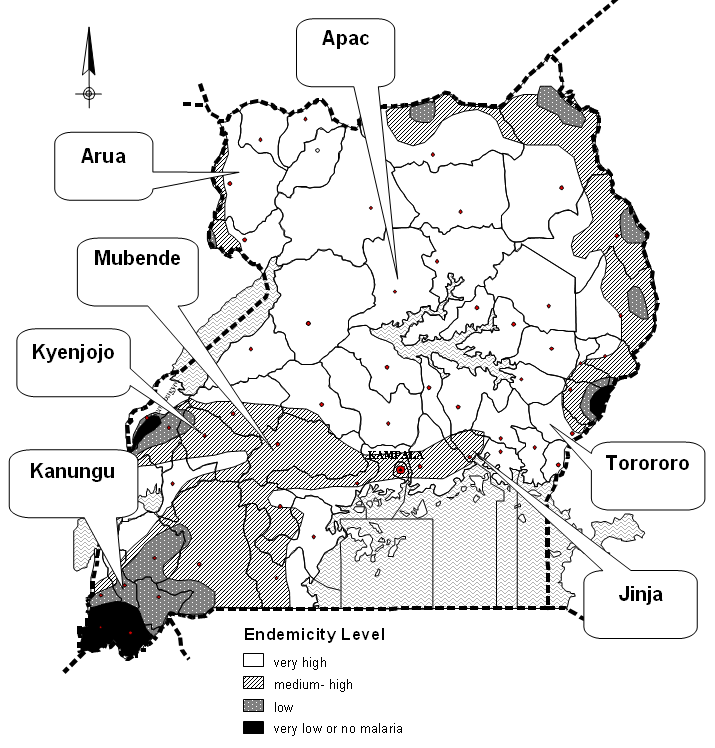
**

- - 1. **Characteristics of sentinel sites and patient populations**

Our seven sentinel sites were chosen to reflect the diversity of malaria around Uganda. Sites vary based on geography and malaria endemicity (Table 1.) Surveillance studies at these sites have benefited greatly from a recent one-year longitudinal entomological study of malaria vectors and their relative contributions to malaria transmission (P. Okello, personal communication). *Anopheles funestus* was the predominant vector at the Apac site, comprising of 92.5 % of the total anopheles mosquitoes collected. *A .gambiae* was the predominant species at the study sites in Tororo, Arua, Jinja, Kyenjojo, Mubende, and Kanungu, where it accounted for 64-91% of the total anophelines collected. Both *A.gambiae* and *A.funestus* were endophagic. The annual *P. falciparum* entomological inoculation rate (EIR) measured in *A. gambiae* and *A. funestus* by enzyme-linked immunosorbent assay was very high, measured at 1,564, 591, and 393 infective bites per person per year (ib/p/y) for the Apac, Tororo and Arua sites, respectively. EIRs were much lower at the Jinja, Mubende, Kyenjojo, and Kanugu sites, measured between 3 and 9 ib/p/y. These measures are, for the most part, consistent with other studies, in which malariometric indices have indicated that northern and eastern Uganda suffer the heaviest burden of malaria (personal communication, Uganda MOH).

Clinical surveillance studies performed by UMSP at sentinel sites (all district health centers) have provided descriptive data on patients diagnosed with malaria (Table 1). These data have shown that malaria disproportionately affects young children under the age of 5 years, especially in the sites with the highest transmission intensity. Areas of high transmission intensity are also associated with a high prevalence of anemia and gametocyte carriage. Reported bednet use varies, but is well below international targets, especially when considering ITNs (insecticide treated nets), which have a reported coverage of 0-12%. These data provide valuable baseline assessments and will complement the surveillance studies outlined in this protocol, as the development of rational strategies to improve malaria control will require an improved understanding of the distribution and determinants of this disease.

Table 1. Characteristics of sentinel sites and patient populations

| **Characteristic** | **Sentinel Sites** | | | | | | |
| --- | --- | --- | --- | --- | --- | --- | --- |
|  | **Kanungu** | **Mubende** | **Kyenjojo** | **Jinja** | **Arua** | **Tororo** | **Apac** |
| Geography  Seasonality | highland unstable | rural  perennial | rural  perennial | urban  perennial | rural  perennial | rural/border  perennial | rural  perennial |
| EIR^*^ | 7 | 3 | 9 | 6 | 393 | 591 | 1564 |
| Age less than 5 years | 79% | 76% | 53% | 61% | 93% | 92% | 90% |
| Parasite density^†^ | 24137 | 20456 | 26017 | 18189 | 23600 | 18438 | 11665 |
| Gametocytes present | 3% | 15% | 23% | 10% | 30% | 11% | 51% |
| Anemia (Hb < 10 gm/dl) | 65% | 59% | 40% | 39% | 63% | 71% | 65% |
| Reported bednet use | 11% | 1% | 4% | 36% | 2% | 24% | 37% |
| Reported ITN use | 3% | 0% | 1% | 5% | 1% | 6% | 12% |
| Recent antimalarial use | 41% | 1% | 19% | 27% | 5% | 7% | 12% |

* Entomological inoculation rate

^†^ Geometric mean

**2.1.6 Results of UMSP antimalarial drug efficacy studies**

The first round of antimalarial drug efficacy studies was completed at all 7 sentinel sites between 2002 and 2004. The primary purpose of these studies was to provide data to help guide policy through standardized evaluation of the safety and efficacy of treatment regimens being considered for first-line use in Uganda. Treatment arms included the MOH recommended combination of CQ + SP, another inexpensive combination of amodiaquine (AQ) + SP, which had been shown to be highly efficacious in studies from Kampala, and the combination of AQ + artesunate (AS), one of the new artemisinin combination therapies recommended by the WHO. These studies enrolled a total of 3,265 patients with over 96% completing the 28 day follow-up period (Table 2).^9,10^ The national regimen, CQ + SP, was highly ineffective, with both high rates of recurrent malaria and treatment failure due to resistant parasites. AQ + SP was associated with moderately high levels of treatment failure due to drug resistant parasites, but had the lowest risk of new infections, likely due to the post-treatment prophylactic effect of these relatively long acting drugs. AQ + AS was associated with the lowest level of drug resistance, but this was counterbalanced by a high risk of new infections, especially at the high transmission sites. Results varied importantly between sites with different malaria endemicities. In general, higher endemicity sites had higher rates of new infections after treatment, but somewhat lower levels of recrudescence, apparently due to improved antimalarial immunity in these populations. These findings illustrate the complex nature of clinical response to antimalarial therapy, highlighting the critical importance of considering variations in malaria endemicity when evaluating the impact of treatment on morbidity, and emphasize the need to continue antimalarial surveillance at multiple sites in Uganda.

In response to the results of these studies and others, the Ugandan government chose to replace CQ + SP with the combination of artemether-lumefantrine (AL) in 2004. AL is now widely supported by the WHO as an appropriate therapy for malaria in Africa, and it has also been adopted by a number of other African countries. A study carried out in a relatively low transmission area (Mbarara, Uganda) showed that AL was highly efficacious, with no treatment failures due to recrudescent parasites and a risk of recurrent malaria over one month of less than 3%.^11^ AL was also highly effective in coastal Tanzania, although reinfections after therapy were common.^12^ Recently, UMSP carried out a comparative study of AL vs. AQ + AS at Tororo, a site with very high levels of malaria transmission. Both treatment arms were associated with a relatively low risk of treatment failure due to recrudescence of resistant parasites. AL was more efficacious than AQ + AS in reducing the risk of recurrent malaria, but, remarkably, even with this more efficacious regimen 49% of patients treated with AL had recurrent malaria by the end of the 28 day follow-up period (Table 2).

Table 2. Results from UMSP drug efficacy studies 2002-2005

| Site | Year | Enrolled | Completed | Recurrent malaria* | | | | Treatment failure^†^ | | | |
| --- | --- | --- | --- | --- | --- | --- | --- | --- | --- | --- | --- |
|  |  |  |  | CQ+SP | AQ+SP | | AQ+AS | CQ+SP | AQ+SP | | AQ+AS |
| Kanungu | 2003 | 367 | 357 (97%) | 85% | 55% | | - | 64% | 33% | | - |
| Mubende | 2003 | 373 | 351 (94%) | 73% | 47% | | - | 37% | 13% | | - |
| Kyenjojo | 2003 | 365 | 349 (96%) | 73% | 40% | | - | 39% | 15% | | - |
| Jinja | 2003 | 543 | 514 (95%) | 63% | 28% | | 19% | 40% | 13% | | 4% |
| Apac | 2003-04 | 542 | 530 (98%) | 66% | 36% | | 52% | 22% | 7% | | 10% |
| Tororo | 2003-04 | 541 | 515 (95%) | 88% | 59% | | 74% | 34% | 18% | | 12% |
| Arua | 2004 | 534 | 522 (98%) | 86% | 53% | | 51% | 46% | 14% | | 9% |
|  |  |  |  | AL | | AQ+AS | | AL | | AQ+AS | |
| Tororo | 2004-05 | 410 | 403 (98%) | 50% | | 66% | | 1% | | 0% | |

* All early treatment failures, late clinical failures, and late parasitological failures

^†^ All early treatment failures and late clinical failures, late parasitological failures due to recrudescence

CQ = chloroquine, AQ = amodiaquine, SP = sulfadoxine-pyrimethamine, AS = artesunate, AL = artemether-lumefantrine

**2.1.7 Current antimalarial drug policy in Uganda**

Minimizing malaria-associated morbidity and mortality is the primary goal of the national policy. To accomplish this, safe, effective, and affordable antimalarial drugs must be provided in a manner that promotes rational drug use and limits the development of drug resistance. The rapidly changing antimalarial drug resistance patterns in Uganda pose a serious challenge to effective treatment. Limited drug efficacy data, a lack of consensus on when to change therapy, and the absence of obvious alternative regimens stagnate policy decision-making.

In 2004, a consensus meeting was held, and there was agreement to abandon CQ + SP as the first-line antimalarial regimen in Uganda. After concerns were voiced about the sustainable efficacy of AQ + SP and AQ + AS, AL was selected to replace CQ + SP. However, at the time this decision was made there were no comparative studies available for this regimen in Uganda, and there were major concerns about cost and availability. Indeed, although the treatment policy has been formally changed, in practice CQ + SP continues to be used. The global shortage of artemisinin combination therapy (ACT), in particular AL, has delayed the implementation of the new Uganda antimalarial drug policy. Uganda has ordered for 15 million doses of AL using funds from the Global Fund for HIV/AIDS, Tuberculosis, and Malaria (GFATM) and the drugs are expected to be in the country next year. Training of health workers on malaria case management using ACT’s is expected to commence late this year. Thus, while an ACT regimen may greatly help in the control of malaria, it is for the most part unavailable, and Ugandans are mostly being treated with CQ or SP monotherapy or CQ + SP, all terribly inadequate regimens. Since the immediate supply of AL and other ACTs is inadequate and long-term availability uncertain, it is critically important that Uganda continues to gather data regarding the efficacies of multiple regimens, including "gold standard" ACT regimens and other, more readily available treatments.

The Ugandan experience with antimalarial drug policy change illustrates the challenges faced by sub-Saharan African countries confronted with rising antimalarial resistance and limited data on potential alternative options. Indeed, twice in the last few years first-line antimalarial regimens were adopted before any data on their efficacy were available from Uganda. Optimal malaria control demands improved surveillance to guide policy. Surveillance of treatment efficacy, collection of additional data on alternative regimens, and active consensus building among key partners in the malaria community will be necessary to develop a rational long-term antimalarial treatment policy in Uganda.

## 2.2 Rationale

ACTs have been strongly advocated for use in Africa, but data on these regimens are currently limited. AL has been chosen as the replacement for CQ+SP as first-line therapy, but data on the efficacy and safety of this regimen are limited in Uganda, and its substantial cost (US$ 2.40 per adult treatment), limited availability, and high reinfection rates after therapy remain significant obstacles. We are proposing to compare the efficacy, safety and tolerability of AL with Dihydroartemisinin-piperaquine (DP) for the treatment of uncomplicated falciparum malaria in Uganda. DP is a fixed-combination antimalarial drug developed in China. Recent randomized clinical trials in Cambodia, Vietnam, and Thailand indicate excellent tolerability and high cure rates against multidrug-resistant falciparum malaria.^13-16^ The data that are collected in this study will be available to the Ugandan MOH to assist in drug policy decision-making.

### 2.3 Study Drugs

### 2.3.1 Artemether-lumefantrine (AL)

AL is a fixed-combination drug containing the artemisinin derivative, artemether, and lumefantrine (previously known as benflumetol). As with other artemisinins, artemether is characterized by rapid antimalarial action, however, recrudescence is frequent when artemether is provided as a single agent, unless given for at least 5-7 days.^17,18^ Lumefantrine also has a high cure rate, but parasite and fever clearance is slower than with artemether.^19^ The fact that AL combines an artemisinin derivative with a novel agent in a fixed-combination regimen is an important advantage of this therapy. It is currently the only co-formulated antimalarial regimen and is included on the WHO Essential Drugs List.^20^ However, AL is administered twice daily (a total of 6 doses over 3 days), and should ideally be taken with fatty food or liquid to maximize absorption, raising concerns about adherence.^21^ Recent studies with AL from Africa have reported encouraging results, with high cure rate and a good safety profile.^11,12,21^ AL has been selected as first-line therapy in several African countries.^22^

**2.3.2 Dihydroartemisinin-piperaquine (DP)**

### DP is a fixed combination drug containing the artemisinin derivative, dihydroartemisinin, and piperaquine. Dihydroartemisinin is highly active and also the main in vivo metabolite of the drugs artesunate and artemether. Piperaquine is a bisquinoline that retains activity against chloroquine-resistant *P. falciparum*. Piperaquine replaced chloroquine as the first-line treatment for malaria in China in 1978. The main side effects of piperaquine have been reported to be nausea, vomiting, and dizziness, which were generally mild and self-limiting.^23^ It is estimated that 4 million Chinese patients were treated annually between 1978 and 1993; a total of 217 metric tons of piperaquine was used.^14^ As a result of mass prophylaxis resistance of *P. falciparum* to piperaquine emerged in the mid-1980’s in China. In the mid-1990’s piperaquine and dihydroartemisinin were studied in various combinations studies in Vietnam (personal communication, Francois Nosten). Recent randomized clinical trials in Southeast Asia have reported excellent tolerability and efficacy against multidrug-resistant *P. falciparum*.^13,15,16^Most relevant to this proposal is a recent randomized controlled trial from Thailand which reported that a simple, once-daily, 3-dose regimen of DP was highly efficacious and safe in children and adults.^14^ This regimen is projected to be relatively inexpensive and has the potential to become an important drug for the treatment of uncomplicated malaria in Africa. In particular, compared to AL it offers decreased cost, simplified dosing, and potentially more reliable drug absorption. However, the antimalarial efficacy of DP in Africa has not yet been reported.

# 3.0 STUDY AIMS

To compare the efficacy, safety, and tolerability of AL and DP for the treatment of uncomplicated falciparum malaria in Uganda.

# 4.0 STUDY DESIGN

## 4.1 Overall study design

The studies will be randomized, single-blinded trials at three sites in Uganda designed according to 2003 World Health Organization (WHO) guidelines for assessment of therapeutic efficacy of antimalarial agents in areas of low, moderate and intense transmission with slight modifications.^24^ The target populations include residents of the catchment areas of the three district health centers where the studies will be performed. The available populations includes residents aged 6 months to 10 years who present to the study clinics with symptoms suggestive of malaria and who have a positive screening thick blood smear. Subjects who meet the selection criteria will be randomized to treatment with one of the two study regimens and will be followed for 42 days. Repeat evaluations will be performed on days 1, 2, 3, 7, 14, 21, 28, 35, and 42 (and any unscheduled day) and will include assessment for the occurrence of adverse events. Treatment efficacy outcomes will be assessed using WHO outcome classification criteria (Appendix 7).

## 4.2 Study outcome and assessment

### 4.2.1 Classification of treatment outcome

Response to treatment will be classified according to the 2003 WHO classification system with slight modifications, and will include adequate clinical and parasitological response (ACPR), early treatment failure (ETF), late clinical failure (LCF), and late parasitological failure (LPF), for purposes of data reporting (Appendix 3). In the final analysis, treatment outcomes will be dichotomized based on the following definitions:

- Recurrent malaria = ETF + LCF
- Recurrent parasitemia = ETF + LCF + LPF.

All ETFs will be considered true treatment failures. For all LCFs and LPFs, molecular genotyping will be used to distinguish recrudescence (true treatment failure) from new infection (see section 11.3).

- Clinical treatment failure = All ETFs + LCFs due to recrudescence
- Parasitological treatment failure = All ETFs + LCFs/LPFs due to recrudescence

### 4.2.2 Primary outcomes

Primary outcomes will be based on the following risks after 42 days of follow-up:

1. Risk of recurrent malaria
2. Risk of recurrent parasitemia
3. Risk of clinical treatment failure
4. Risk of parasitological treatment failure

Risks will be estimated using the Kaplan-Meier product limit formula based on a modified intention-to-treat analysis (see section 9.2).

### 4.2.3 Secondary outcomes

1. Prevalence of fever (defined as both subjective fever in the previous 24 hours and measured axillary temperature > 37.5°C) on follow-up days 1, 2, and 3.
2. Prevalence of parasitemia on follow-up days 2 and 3.
3. Change in mean hemoglobin from day 0 to 42 (or day of rescue therapy for patients classified as LCF or LPF)
4. Prevalence of gametocytemia and gametocyte density on follow-up days 2, 3, 7, 14, 21, 28, 35, and 42.
5. Risk of serious adverse events: proportion of patients experiencing any serious adverse event in each treatment group during the 42-day follow-up period (both including and excluding patients classified as ETF or LCF, as recurrent malaria can be confounding).
6. Risk of adverse events of moderate or greater severity, at least possibly related to the study medications (both including and excluding patients classified as ETF or LCF).
7. Change in the prevalence of molecular markers associate with drug resistance from day 0 to the day of recurrent parasitemia.

### 4.3 Randomization:

Computer generated randomization lists will be created for each of the 3 study sites by a member of the project who will not be directly involved in the conduct of the study. Sealed copies of the original randomization lists and documentation of the procedure used to generate the lists will be stored in the project administrative offices in Kampala. Prior to the onset of the study, sealed copies of the randomization lists will be distributed to the study nurse responsible for treatment allocation.

# 5.0 PARTICIPANT SELECTION AND ENROLLMENT

## 5.1 Study sites

The study will be conducted at 3 of the Uganda Malaria Surveillance Project (UMSP) sentinel sites that were originally established in 1998 by the Ugandan Ministry of Health (MOH) in collaboration with the East African Network for Monitoring Antimalarial Treatment (EANMAT).

**Table 3. UMSP sentinel sites**

| **District** | **Health center** | **Region type** | **Catchment population** | **Transmission** |
| --- | --- | --- | --- | --- |
| Apac | Aduku | Rural | 44,000 | Holoendemic |
| Kanungu | Kihihi | Rural | 14,000 | Mesoendemic |
| Mubende | Kasambya | Rural | 40, 000 | Mesoendemic |

## 5.2 Malaria surveillance, screening and recruitment process (Appendix 1)

Study subjects will be recruited from the OPD (outpatient department) of the sentinel health centers. Patients who present with symptoms suggestive of malaria (fever or history of fever) will be referred to the outpatient laboratory for a fingerprick for screening thick blood smear (using standard Giemsa staining) and malaria rapid diagnostic tests (RDTs). Thick blood slides will be read and counted by the laboratory technicians. The parasite density of positive screening thick blood smears will be estimated by the laboratory technicians by counting the number of asexual parasites per 200 leukocytes, assuming a leukocyte count of 8,000/μl. At the time of screening, study personnel will record in a log basic information including patient age, gender, the date, and results of the thick smear and RDTs. All patients who have a positive screening thick smear with a parasite density of > 2000/ul (> 50 parasites per 200 leucocytes) and < 200,000/ul (< 5000 parasites per 200 leucocytes) will be referred to the study clinic for further evaluation. If the patient satisfies the selection criteria, they will be enrolled in the study. All patients who do not satisfy the selection criteria and are excluded from study enrolment will be referred back to the outpatient department for appropriate care. RDTs are being assessed as a surveillance tool for malaria at the district health centers; RDT results will not impact upon patient care.

## 5.3 Selection criteria

On day 0, patients with symptoms suggestive of malaria and a positive screening thick blood smear will be assessed for the following selection criteria by study physicians:

1. Not previously enrolled in this study
2. Age 6 months – 10 years
3. Weight > 5 kg
4. Fever (> 37.5ºC axillary) or history of fever in the previous 24 hours
5. Absence of any history of serious side effects to study medications
6. No evidence of a concomitant febrile illness in addition to malaria
7. Provision of informed consent (appendix 5) and ability to participate in 42-day follow-up (patient has easy access to health unit)
8. No danger signs or evidence of severe malaria defined as:

- Unarousable coma (if after convulsion, > 30 min)
- Repeated convulsions (> 2 within 24 h)
- Recent convulsions (1-2 within 24 h)
- Altered consciousness (confusion, delirium, psychosis, coma)
- Lethargy
- Unable to drink or breast feed
- Vomiting everything
- Unable to stand/sit due to weakness
- Severe anemia (Hb < 5.0 gm/dL)
- Respiratory distress (labored breathing at rest)
- Jaundice

Patients fulfilling these criteria will be assigned a study number and will be referred to the laboratory. A second fingerprick blood sample will be obtained to prepare thick and thin blood smears, collect blood on filter paper, and for measurement of hemoglobin. Patients with hemoglobin levels < 5.0 gm/dL will be excluded based on criteria for severe malaria (see above) and immediately referred back to the study physicians for appropriate care. After going to the laboratory, the subjects will be referred to the study nurse for treatment allocation and treatment with the study medications. Patients must also meet the following criterion:

1. Absence of repeated vomiting of study medications on day 0

Results of the Giemsa-stained thick and thin blood smears obtained on day 0 will not be available until after the patients have been treated and discharged from the clinic. Patients will return to the clinic on day 1 and will be excluded from the study if the following inclusion criteria are not met:

1. *P. falciparum* mono-infection
2. Parasite density > 2000/ul and < 200,000/ul

# 6.0 BASELINE EVALUATION AND TREATMENT ALLOCATION

## 6.1 Baseline evaluation and procedures

On day 0, patients fulfilling the selection criteria will be assigned a study number and will undergo a complete history and physical examination (Appendix 9). Patients will be referred to the laboratory to obtain a fingerprick blood sample for repeat thick blood smear, thin blood smear, hemoglobin measurement, and to save 4 blood spots on filter paper for future molecular testing. After going to the laboratory, the patients will be referred to the study nurse for treatment.

## 6.2 Treatment group assignment

Patients will be randomly assigned to one of the two treatment groups (AL or DP). Randomization will be done according to a pre-determined randomization list. The randomization list will be computer generated by a member of the project who will not be directly involved in the conduct of the study. The randomization list will include consecutive treatment numbers with corresponding random treatment assignments. A separate randomization list will be prepared for each study site. Prior to starting the studies at each site, a project administrator (not involved in the conduct of the study) will print a copy of the treatment randomization list and place it in a sealed envelope to be delivered to the study site. Only the study nurse will have access to the sealed treatment randomization list. At the study sites, treatment allocation and administration of medications will be performed by the study nurse. A sample of the treatment randomization list is included below:

**Sample treatment randomization list**

| **Treatment number** | **Assigned treatment** | **Study number** | **Date of treatment assignment** | **Study drugs administered** | | |
| --- | --- | --- | --- | --- | --- | --- |
|  |  |  |  | Day 1 | Day 2 | Day 3 |
| 1 | AL |  |  |  |  |  |
| 2 | AL |  |  |  |  |  |
| 3 | DP |  |  |  |  |  |
| 4 | AL |  |  |  |  |  |
| 5 | DP |  |  |  |  |  |

**6.3 Treatment allocation**

At the time the first patient is enrolled in the study, the study nurse will break the seal of the envelope containing the treatment randomization list. To allocate subjects to the appropriate treatment group, the study nurse will select the next available treatment number and corresponding study regimen. The study nurse will record the date and time of treatment assignment and the patient's study number. The study nurse will also record when each dose of study medication is given. Only the study nurse will have access to the treatment randomization list and the assigned treatments throughout the conduct of the study. At the completion of the studies at each site the treatment randomization list and assigned treatments will be placed in a sealed enveloped and returned to the project administer not involved in the conduct of the study. Un-blinding of the study will only occur at the time data entry and cleaning are completed for each study or at the request of the Drug Safety Monitoring Board.

## 6.4 Study drugs

**Table 4. Drug formulation and labeling**

| **Drug** | **Trade name (Manufacturer)** | **Class** |
| --- | --- | --- |
| Artemether-lumefantrine  (20mg/120 mg) | Coartem  (Novartis) | artemisinin derivative + dibutyl-aminoethanol |
| Dihydroartemisinin-piperaquine (40mg/320mg) | Duo-Cotecxin  (Holley-Cotec Pharmaceuticals) | Artemisinin derivative + bisquinoline |

### 6.4.1 Dosing of study drugs

All subjects will receive one dose of medication in the morning and one dose of medication in the evening for 3 days. Subjects randomized to the AL group will receive AL twice daily for 3 days, given in fixed dose tablets (20 mg artemether + 120 mg lumefantrine) according to weight-based guidelines established by the manufacturer (Novartis). Subjects randomized to the DP group will receive DP once daily for 3 days in the morning given in fixed dose tablets (40 mg dihydroartemisinin + 320 mg piperaquine) according to weight-based guidelines and placebo tablets once daily for 3 days in the evening. A weight-based regimen consisting of a total dose of 6.4 and 51.2 mg/kg of dihydroartemisinin and piperaquine, respectively, will be given in 3 equally divided daily doses rounded up or down to the nearest quarter tablet.^14^ Dosing of placebo tables will mimic that of DP. Details of the dosing schedule are included in Appendix 6.

## 6.4.2 Blinding

Study medications will be administered according to weight-based guidelines (Appendix 6). Study medications will not be identical in appearance or taste, but the number of doses received will be the same (1 medicine in the morning and 1 medicine in the evening) for patients in both treatment groups. Patients will not be informed of their treatment regimen, and all study staff involved in the assessment of patient outcomes, including the study clinicians (responsible for clinical assessment and measurement of temperature) and laboratory technicians (responsible for reading thick blood smears and determining parasite density) will be blinded to the treatment group assignments

### 6.4.3 Administration of study drugs

Administration of all study drugs will be done in the clinic and directly observed. The first dose of study medication on day 0 will be administered before noon and the second dose administered 8 hours later. The first dose of study medication on days 1 and 2 will be administered before 10 am and the second dose administered 8-10 hours later. The patient will have the option of remaining at the clinic for their second dose of medication or going home and returning to the clinic later in the day. For patients who choose to go home between doses, a home visitor will collect information on where they live. If a patient fails to return to the clinic in a timely manner for their second daily dose of study drug, they will be visited at home and brought to the clinic the same day. If patients miss their second dose of study drug, they will be excluded from the study (see section 7.3). The study nurse will record the date and time study drugs are administered. Study drugs given to young children will be crushed, mixed with water, and administered as a slurry. Study drugs administered to older children will be given as tablets or fractions of tablets to be taken orally with a glass of water. Patients will also be given a small glass of milk after each dose of study medication in accordance with recommendations to improve the absorption of AL. The study nurse will directly observe consumption of study drug. Patients will be observed for 30 minutes to ensure that the medications are not vomited. Any patient who vomits the medication within 30 minutes of administration will be retreated with a second dose. Any patient who vomits repeatedly (> 3 times) will be treated with or referred for treatment with parenteral quinine and recorded as an early treatment failure based on fulfilling criteria for dangers signs.

## 6.5 Additional medications

On day 0, patients will receive paracetamol (10mg/kg) to take every 8 hours until the resolution of fever. Patients found to have uncomplicated malaria and a concomitant illness will be treated for both and followed up according to the study protocol. For patients with anemia (Hb < 10 gm/dL), we will follow Integrated Management of Childhood Illness (IMCI) guidelines: anemic children will be treated with iron sulfate (100 mg daily for 2 weeks) and mebendazole (only children > 1 year of age; 250 mg age 1-2 years; 500 mg > 2 years age; treated no more frequently than every 6 months).

# 7.0 FOLLOW-UP EVALUATION AND PROCEDURES

## 7.1 Follow-up schedule

Patients will be asked to return to the clinic for follow-up on days 1, 2, 3, 7, 14, 21, 28, 35, 42, and any unscheduled day that they feel ill. All patients will be reimbursed the cost of their transport to and from the clinic. At enrollment, details about the location of the patient’s residence will be obtained and if a subject does not return for a scheduled clinic follow-up appointment, the study personnel shall visit them at home. Patients who return on day 1 and fail to fulfill the criteria of *P. falciparum* mono-infection with a parasite density of > 2000 parasites/ul and < 200,000 parasites/ul will be excluded from further study and referred to OPD for management. At each repeat visit, temperature will be measured and a focused physical examination will be performed. A finger prick blood sample will be obtained on days 2, 3, 7, 14, 21, 28, 35, 42, and any extra day they present with a fever to repeat thick blood smears and to save blood on filter paper. Hemoglobin will be re-evaluated on day 42 or at the time of clinical treatment failure (ETF or LCF).

Table 5. Follow-up Schedule

|  | **Day 0** | **Day 1** | **Day 2** | **Day 3** | **Day 7** | **Day 14** | **Day 21** | **Day 28** | **Day**  **35** | **Day**  **42** | **Extra**  **Day** |
| --- | --- | --- | --- | --- | --- | --- | --- | --- | --- | --- | --- |
| **Study drugs** | X | X | X |  |  |  |  |  |  |  |  |
| **History** | X | X | X | X | X | X | X | X | X | X | X |
| **Temperature** | X | X | X | X | X | X | X | X | X | X | X |
| **Physical exam** | X | X | X | X | X | X | X | X | X | X | X |
| **Thick blood smear** | X |  | X | X | X | X | X | X | X | X | X |
| **Thin blood smear** | X |  |  |  |  |  |  |  |  |  |  |
| **Filter paper sample** | X |  | X | X | X | X | X | X | X | X | X |
| **Hemoglobin** | X | † | † | † | † | † | † | † | † | X | † |
| **Assessment for AEs** | X | X | X | X | X | X | X | X | X | X | X |

X = perform this task

† Performed only if clinical treatment failure

## 7.2 Classification of treatment outcome and management of treatment failure

Patients will be followed for 42 days and will have treatment outcomes assessed according to the WHO 2003 guidelines with slight modifications (Appendix 7). Patients who meet criteria for ETF, LCF, or LPF will be started on rescue therapy with quinine 10 mg/kg orally three times a day for 7 days. Any patient diagnosed with severe malaria or danger signs during follow-up, will be referred for appropriate treatment with parenteral quinine at the local facility or hospital. Patients not admitted to the hospital will receive oral quinine to complete a 7-day course of therapy. If a patient requires rescue therapy, formal study follow-up will end, and the patient will be followed up at the discretion of the study physician. Quinine is currently the recommended therapy for those who fail initial antimalarial treatment in Uganda.

## 7.3 Exclusion after enrollment and loss to follow-up

Patients will be excluded from further study participation and not have their treatment outcome assessed if any of the following occurs after enrollment:

- 1. Use of antimalarial drugs outside of the study protocol.
  2. Development of a febrile illness (e.g. pneumonia, dysentery, measles) concomitantly with parasitemia which interferes with outcome classification.
  3. Withdrawal of informed consent.
  4. Loss to follow-up: Patients who fail to receive all of their doses of study medication on days 0-2 and those who fail to attend a follow-up visit and are unable to be located within 24 hours on Day 3 or within 48 hours on Days 7-42.

Patients who are excluded from further study participation after enrollment will still be eligible to receive standard medical care at the study clinic.

## 7.4 Management of non-malarial illnesses

Patients who are found to have illnesses other than malaria during standard 42-day follow-up will receive standard-of-care treatment in the clinic, according to standardized algorithms, or will be referred to OPD for the appropriate care. Routine use of non-study medications with antimalarial activity, including tetracycline, antifolate, and macrolide antibiotics, will be avoided when acceptable alternatives are available.

**7.5 Distribution of insecticide treated bednets (ITNs)**

All patients enrolled in the studies will be given a free long-acting ITN on Day 0 with instructions on its proper use. ITNs are being distributed as part of a program to decrease the risk of new infections following treatment for malaria.

# 8.0 ASSESSMENT FOR ADVERSE EVENTS

## 8.1 Definitions

An adverse event is defined as "any untoward medical occurrence in a patient or clinical investigation subject administered a pharmaceutical product that does not necessarily have a causal relationship with this treatment" (ICH Guidelines E2A). An adverse event can further be broadly defined as any untoward deviation from baseline health which includes:

- Worsening of conditions present at the onset of the study
- Deterioration due to the primary disease
- Intercurrent illness
- Events related or possibly related to concomitant medications

(International Centers for Tropical Disease Research Network Investigator Manual, Monitoring and Reporting Adverse Events, 2003).

A serious adverse event is defined as an experience that results in any of the following outcomes:

- Death during the period of study follow-up
- Life-threatening experience (one that puts a patient at immediate risk of death at the time of the event)
- Inpatient hospitalization during the period of study follow-up
- Persistent or significant disability or incapacity
- Specific medical or surgical intervention to prevent one of the other serious outcomes listed in the definition.

## 8.2 Identification of adverse events

At each follow-up visit (days 1, 2, 3, 7, 14, 21, 28, 35, 42, and any unscheduled day), study clinicians will assess patients according to a standardized clinical record form (appendix 12). A severity grading scale, based on toxicity grading scales developed by the WHO and the National Institutes of Health, Division of Microbiology and Infectious Diseases, will be used to grade severity of all symptoms, physical exam findings, and hemoglobin results (Appendices 9). Any new event, or an event present at baseline that is increasing in severity, will be considered an adverse event.

## 8.3 Reporting of adverse events

For each possible adverse event identified and graded as moderate, severe or life threatening, an adverse event report form will be completed (Appendix 12). An adverse event report form will not be completed for events classified as mild as these symptoms are common and difficult to distinguish from signs and symptoms due to malaria. The following information will be recorded for all adverse experiences that are reported:

1. Description of event
2. Date of event onset
3. Date event reported
4. Maximum severity of the event
5. Maximum suspected relationship of the event to study medication
6. Is the event serious?
7. Initials of the person reporting the event
8. Was the event episodic or intermittent in nature?
9. Outcome
10. Date event resolved

## 8.4 Reporting of serious adverse events

Guidelines for reporting of serious adverse events provided by the UCSF Committee for Human Research, Makerere University IRB, and the Ugandan National Council for Science and Technology will be followed (see section 13.4).

# 9.0 STATISTICAL CONSIDERATIONS

## 9.1 Sample size calculations

The sample size calculations will be made for the studies at each individual sites based on estimated risks of recurrent parasitemia (outcomes classified as ETF, LCF, or LPF) of the treatment regimens to be studied. If the null hypothesis states that there is no difference in risk of recurrent parasitemia between the two treatment groups, we plan to test the alternative hypothesis that the risk of recurrent parasitemia will differ between the two treatment groups. We estimate that the risk of recurrent parasitemia after 42 days will be 50% in Kanungu and Mubende and 66% in Apac based on previous UMSP studies. We plan to enroll 200 patients in each treatment arm at all of the study sites (400 patients per site, 800 patients total). Based on an α = 0.05 (two-sided) and a power of 80%, allowing for 10% loss to follow-up, we will have the ability to detect a risk difference of 15% between the two treatment groups in Kanungu and Mubende and 15% between the two treatment groups in Apac.

## 9.2 Analytical plan

Data analysis will be primarily performed by the project epidemiologist using SPSS and STATA statistical software packages, with additional assistance from collaborators at UCSF. Descriptive statistics will be used to summarize baseline characteristics of study patients. Efficacy and safety data will be evaluated using a modified intention-to-treat analysis and will only include patients who meet all selection criteria. Because final selection criteria are assessed on Day 1 after final reading of the enrollment thick and thin smears, some patients randomized to treatment but not fulfilling selection criteria will be excluded from the modified intention-to-treat analysis. Categorical variables will be compared between the treatment groups using chi-square tests or Fisher’s exact tests and continuous variables will be compared using t-tests or non-parametric tests. A p-value of < 0.05 will be considered statistically significant. Estimates of the risk of failure for all primary outcomes will be made using the Kaplan-Meier product limit formula. Patients excluded after enrollment will be censored at the time of their last assessment. Additionally, for genotyping adjusted outcomes, patients with recurrent malaria or recurrent parasitemia due to new infections will be censored.

# 10.0 DATA COLLECTION AND MANAGEMENT

## 10.1 Data management

All clinical data will be recorded onto standardized case record forms by study clinicians. Laboratory data will be recorded in a laboratory record book by the study laboratory technicians and then transferred to the case record forms by the study clinicians. Data will be transferred from the case record forms into a computerized database (EPI INFO 6.04) by data entry personnel and will be double entered to verify accuracy of entry. Two back-up files of the database will be stored on compact discs after each data entry session. For quality control, check programs will be written into the database to limit the entry of incorrect data and ensure entry of data into required fields.

## 10.2 Data quality assurance and monitoring

All members of the study team will be educated in the study protocol prior to the onset of the trial. The study clinicians will complete case record forms at each patient visit. These forms will be reviewed by the study coordinator and site supervisors from the core facility for completeness and accuracy. For quality control of thick blood smear slide readings, expert microscopists who will be blinded to the patient’s treatment group will repeat the reading of all slides. All discrepant slide readings will be resolved based on the results of a 3^rd^ reading. Study group meetings will be conducted by the coordinator once a week to assess progress of the study, address any difficulties, and provide performance feedback to the members of the study group. In addition members from the core facility will make regular visits to active study sites as needed.

## 10.3 Records

Case record forms will be provided for each subject. Participants will be identified by their initials and study identification number on the case record form. Patient names will not be entered into the computerized database. All patient record forms will be kept in individual files in a secure filing cabinet in the study clinic. All corrections will be made on case record forms by striking through the incorrect entry with a single line and entering the correct information adjacent to it. The correction will be initialed and dated by the investigator. Any requested information that is not obtained as specified in the protocol will have an explanation noted on the case record form as to why the required information was not obtained. Additional records will be kept in the clinical and laboratory record books at the core facility in Kampala. The investigators will allow all requested monitoring visits, audits or reviews.

# 11.0 LABORATORY PROCEDURES

## 11.1 Blood smears

Screening thick smears will be stained with 10% Giemsa for 10 minutes. Study thick and thin blood smears will be stained with 2% Giemsa for 30 minutes. Thick blood smears will be evaluated for the presence of parasitemia (asexual forms only) and gametocytes. Parasite and gametocyte densities will be calculated from thick blood smears by counting the number of asexual parasites and gametocytes, respectively, per 200 leukocytes (or per 500, if the count is <10 parasites or gametocytes/200 leukocytes), assuming a leukocyte count of 8,000/μl. A thick blood smear will be considered negative when the examination of 100 high power fields does not reveal asexual parasites or gametocytes. Thin blood smears will be evaluated to determine parasite species.

## 11.2 Hemoglobin measurement

Hemoglobin will be measured from fingerprick blood samples using a portable spectrophotometer (HemoCue, Anglom, Sweden).

## 11.3 Molecular studies

Blood samples will be collected from patients on days 0, 2, 3, 7, 14, 21, 28, 35, 42 and on any unscheduled day that the patient presents with clinical deterioration or recurrent fever. Blood will be placed onto filter paper in approximately 25 ul aliquots per blood spot (4 blood spots per sample). The samples will be labeled, air-dried and stored in small, sealed sample bags at ambient temperature. Parasite DNA will subsequently be removed from the filter paper and prepared for molecular analysis using a chelex extraction method. Genotyping will be performed on all patients with outcomes classified as LCF or LPF. Genotyping of parasites collected at baseline (day 0) and the day of recurrent parasitemia will be done to distinguish between true recrudescence and new infections. Briefly, selected regions of the merozoite surface protein-2 gene, merozoite surface protein-1 gene, and 6 microsatellite markers will be amplified using PCR and characterized based on sequence and size polymorphisms identified by gel electrophoresis. Genotyping patterns on the day of recurrent parasitemia will be compared with those at treatment initiation using GelCompar II software (Applied Maths). This laboratory work will be performed in Kampala or at UCSF.

# 12.0 PROTECTION OF HUMAN SUBJECTS

## 12.1 Institutional Review Board (IRB) review and informed consent

This protocol and the informed consent documents, including any additional educational or recruitment material, will be reviewed and approved by the Makerere University IRB, Ugandan National Council of Science and Technology, and the UCSF Committee for Human Research before the trial begins. Any amendments or modifications to this material will be reviewed and approved by these bodies prior to implementation.

## 12.2 Risks and discomforts

### 12.2.1 Privacy

Care will be taken to protect the privacy of subjects, as described in this protocol. However, there is a risk that others may inadvertently see patients’ medical information, and thus their privacy may be compromised.

### 12.2.2 Risks of randomization

This will be a randomized trial, and some treatment arms may prove to be more or less efficacious, more or less well tolerated, and/or more or less safe than others. Thus, there is the risk that patients will be randomized to less efficacious, less well tolerated, and/or less safe treatment regimens.

### 12.2.3 Fingerprick blood draws

Risks include pain, transient bleeding and soft-tissue infection.

### 12.2.4 Risk of artemisinins

Artemisinin derivatives have now been extensively studied, and they are remarkable for a lack of serious toxicity when used for the treatment of malaria.^25^ Considering all artemisinins, 15% (12,463) of the patients enrolled in all published antimalarial drug trials over the past 50 years have received an artemisinin compound, and there are more trials on these compounds than on any other antimalarials (N. White, unpublished communication). In addition to formal studies, artemisinins have now been widely used, with well over a million treatments, mostly of artesunate (AS), in Southeast Asia. The only serious toxicity which has emerged in detailed prospective clinical evaluations is a low risk of type 1 hypersensitivity reactions (estimated risk 1:2833, 95% CI 1:1362-1:6944).^26^ Electrocardiograms and detailed neurological, audiometric, and neurophysiological tests have failed to show any evidence for cardiac or neurological toxicity in humans (see below for more details).^27-29^

Animal studies have led to some concerns over artemisinins, particularly regarding cardiac and neurological effects, and reproductive toxicity. As slight QT prolongation was observed in dogs treated with high doses, detailed electrocardiographic studies have been conducted in humans during treatment for falciparum malaria.^28-32^ Taking into account effects of malaria, no significant effects of artemisinins on the QT interval were identified.

The neurological effects of artemisinins have been very extensively studied. In mice, rats, dogs, and monkeys, high dosages of intramuscular artemether and arteether produce an unusual and selective pattern of damage to certain brainstem nuclei, particularly those of the auditory and vestibular systems.^33-43^ AS is transformed in vivo to dihydroartemisinin, which is the most neurotoxic of the artemisinin derivatives.^44-46^ However, in the animal models, orally administered AS and dihydroartemisinin are considerably less neurotoxic than intramuscular artemether or arteether. Differences in toxicity are explained by differences in pharmacokinetics of different compounds and different routes of administration.^39,41,42,47^ Neurotoxicity results from the long-lasting blood concentrations that follow intramuscular injection of the oil-soluble compounds, artemether and arteether. Oral administration of artemether or arteether, which provides much more rapid absorption and elimination than intramuscular dosing, leads to markedly less neurotoxicity in mice, although oral artemether can be made more neurotoxic by giving the drug in small repeated doses to simulate the constant exposure that follows intramuscular injection.^39^ Artesunate is much less toxic than arteether in rats when administered intramuscularly ^36^ or orally.^40,41,48^ Importantly, with high dose intramuscular injections of artemether and arteether, clinical assessment of mice was a sensitive indicator of neurotoxicity; no mice with normal clinical exams showed histopathology.^45^

The artemisinin derivatives are remarkably well tolerated in humans. In a clinical safety review of 108 studies including 9,241 patients, no serious adverse events or significant toxicity was reported.^49^ In addition, a systematic review of artemisinin derivatives for treating uncomplicated malaria, including 41 studies of 5,240 patients, showed no evidence of harmful effects related to artemisinin derivatives.^50^ Clinical studies have shown no convincing evidence for neurotoxicity after treatment with artemisinin derivatives, though neurological effects of acute malaria are common. One letter described ataxia and slurred speech after AS therapy, but these findings were consistent with the course of severe malaria.^51^ To specifically evaluate for potential artemisinin-associated auditory toxicity in humans, van Vugt et al. performed clinical neurological evaluations, audiometry and early latency auditory evoked responses in 79 patients treated with multiple doses of artemether or artesunate and 79 matched controls in Thailand, and no evidence of auditory toxicity was detected.^29^ Comparisons of patients who had received multiple courses of artemisinin derivatives with age-matched untreated controls showed no significant differences in clinical, audiometric, or auditory evoked potential measurements.^27,29^ Even considering the most worrisome dosing regimen, there is no evidence that clinical use of intramuscular artemether has caused neurotoxicity. In a new report, four independent neuropathologists examined the brains of patients who died after treatment with intramuscular artemether, and there was no evidence for the characteristic pattern of neuropathological change seen in the animal studies.^52^ These results suggest a wide margin of safety for artemisinins in clinical use, particularly when given orally, particularly for water soluble compounds, and most particularly for the most widely studied water-soluble agent, AS.

### 12.2.5 Risk of artemether-lumefantrine (AL)

AL (Coartem; Novartis) has been extensively studied through Good Clinical Practice (GCP) standardized preclinical and clinical trials and was added to the WHO Essential Medicines List in 2002.^53^ It has been approved for use against malaria in both developing and developed (e.g. Switzerland) countries. The drug appears to be very well tolerated, especially in comparison to other antimalarials and antimalarial combinations including chloroquine, quinine, and mefloquine+artesunate. A clinical safety review of children under 12 years of age showed that the most common adverse events were abdominal pain, cough, anorexia, headache, vomiting, and diarrhea (all seen in 5-12% of subjects, Novartis, Coartem monograph, 3^rd^ ed. 2004).

An integrated review of toxicity in 1869 patients (611 under age 13) showed the most commonly reported adverse events were gastrointestinal disturbances (abdominal pain, anorexia, nausea, vomiting diarrhea), headache, and dizziness. Rash and pruritis were reported in <2% of patients. No serious or persistent neurological toxicities were linked to AL therapy. Of 20 severe adverse events in 1869 patients, 19 were likely attributable to underlying malaria or concomitant illness, and one was possibly related to AL use (hemolytic anemia in a 35-year-old 13 days after the last administered dose).^54^ One concern addressed in studies of AL was possible cardiac arrhythmogenic potential, based on similarities in the chemical structures of lumefantrine and halofantrine. Halofantrine can cause defects in cardiac conduction, particularly a marked QT prolongation that can produce arrhythmias. In 713 patients treated with lumefantrine and followed with serial electrocardiograms, no adverse clinical cardiac events were recorded. Although trials have been limited to date, no serious cardiotoxicity or neurotoxicity has been reported with the use of AL.^19,21,29,55^

### 12.2.6 Risk of dihydroartemisinin-piperaquine (DP)

### DP is an artemisinin-containing fixed-combination drug developed in China. Recent randomized clinical trials in Cambodia, Vietnam, and Thailand indicate excellent tolerability and high cure rates against multi-drug resistant falciparum malaria. Artemisinin derivatives such as dihydroartemisinin have been used safely in large numbers of patients with uncomplicated or severe malaria as detailed in section 12.2.4. Piperaquine has been used less widely, but it has been a standard antimalarial drug in China since the 1970s. Data from in vitro and animal studies suggest that it is as potent as chloroquine but less toxic.^56-59^ In the first human studies of piperaquine, the main side effects were mild headache, listlessness, nausea, and dizziness.^60^ In a study of the safety and efficacy of DP in 106 Cambodian children and adults with uncomplicated malaria, adverse events were uncommon (< 5%), mild, short lived, and difficult to distinguish from symptoms of malaria (anorexia, nausea, vomiting, abdominal pain, diarrhea, and dizziness). ^15^In a safety evaluation of DP in 62 Cambodian children and adults with malaria, DP was found to be safe and well tolerated with no evidence of clinically significant postural hypotension, QTc prolongation, or propensity for hypoglycemia.^61^In a clinical trial of DP in 166 Vietnamese patients with uncomplicated malaria, 3% of patients reported minor adverse events, mostly transient nausea, which were self limited and resolved with the abatement of fever.^16^ In a dose-optimization clinical trial of DP in 487 children and adults from Thailand with uncomplicated malaria, DP was well tolerated, with a low incidence of mild adverse events, which were mainly upper gastrointestinal and were similar to those reported in other studies.^13^ In a clinical trial of DP in 331 children and adults from Thailand with uncomplicated malaria, DP was well tolerated, with a low incidence of mild side effects and no serious adverse events felt to be related to the study drug.^14^ DP is now in routine use in Vietnam with no reports of serious adverse events (although limited resources are available for pharmacovigilance).

### 12.2.7 Risk of Quinine

Quinine is the standard drug for the treatment of severe malaria throughout Africa and is also the standard drug for the treatment of falciparum malaria in the U.S. It will be the treatment for patients who fail therapy with any study regimen. Quinine can commonly cause tinnitus, headache, nausea, dizziness, flushing, and visual disturbances. These symptoms, termed “cinchonism”, do not warrant discontinuing therapy unless they are severe. Less common toxicities include vomiting, diarrhea, and abdominal pain. Rare toxicities include skin rashes, urticaria, angioedema, bronchospasm, and hematologic abnormalities (hemolysis, leukopenia, agranulocytosis, and thrombocytopenia). Quinine can cause hypoglycemia, especially in pregnancy. Cardiovascular toxicity is seen principally with intravenous quinine, which will not be used in this study.

## 12.3 Compensation

The patients/patients’ families will receive reimbursement for transportation costs to and from the clinic. In addition, all clinic visits, antimalarial medication, and the evaluation and treatment for some routine medical problems encountered during follow-up will be provided free of charge. Medical care that the patient receives which is unrelated to malaria will remain the primary responsibility of the patient, parent or guardian, although routine medical problems will generally be managed by the study at no cost to the patient.

## 12.4 Consent procedures

All screening interviews will be conducted in the native language of the patients by the study personnel (with a translator if necessary). Consent forms will be provided to the parents or guardians for their review. Study clinicians will seek formal consent. The parents or guardians will be asked to sign consent to participate in the research study. The informed consent will describe the purpose of the study, the procedures to be followed, and the risks and benefits of participation. If a parent or guardian is unable to read or write, his/her fingerprint will be used in substitute for a signature, and a signature from a witness to the informed consent discussion will be obtained. Parents or guardians will be informed that participation of their child(ren) in the study is completely voluntary and that they may withdraw from the study at any time.

## 12.5 Alternatives

Individuals whose parents or guardians choose not to participate in this study will not be enrolled. They will receive standard care for medical problems as they arise at the government health dispensaries or other medical facilities in the UMSP sentinel sites.

## 12.6 Confidentiality of records

Patients, parents and guardians will be informed that participation in a research study may involve a loss of privacy. All records will be kept as confidential as possible. Patients will be identified primarily by their study number and patient names will not be entered into the computerized database. No individual identities will be used in any reports or publications resulting from the study.

**13.0 CLINICAL AND SAFETY MONITORING.**

**13.1 Data and safety monitoring board**

A data and safety monitoring board will be assembled, consisting of a chairman, one statistician, and one clinician.

**13.2 Monitoring plan**

An interim report will be prepared when approximately one-half of the total projected sample size has been recruited (200 patients). The interim report will contain information on study progress and data quality (including subject recruitment, patient follow-up, and protocol adherence), safety data (serious adverse events). In addition, the clinical monitor will be asked to review any serious adverse events identified during the study.

**13.3 Stopping guidelines**

Interpretation of results and decisions about discontinuation of the study will be made by the members of the DSMB. Interim/cumulative data for evidence of efficacy will not be provided as we do not believe that there will be grounds for stopping the study on the basis of drug efficacy.

**13.4 Reporting of serious adverse events from sentinel sites to Kampala**

When a serious adverse event (SAE) occurs, the medical officer at the sentinel site shall call the Kampala core facility staff on the Adverse Event Reporting hotline within 24 hours of discovering the event. Kampala core facility staff will complete a SAE Data Collection Form and prepare a Serious Adverse Event Form – Initial Report. The Kampala core facility staff will be responsible for notifying the IRBs that a serious adverse event has occurred. All serious events shall be reported to the UCSF Committee of Human Research (CHR) within 10 days of the onset of the event. A UCSF AE report form (one for each event) shall be completed and faxed along with copies of the UMSP Serious Adverse Event Form – Initial Reports to the CHR. Any protocol deviations or problems involving study conduct or patient participation shall also be reported in a similar manner.

Quarterly reports of all serious AEs will be submitted to the Executive Secretary of NDA and the UNCST. The reports shall include a summary list of all serious AEs occurring during the quarter and copies of all UMSP Serious Adverse Event Forms, including both the Initial Reports Forms (one for each event) and Follow-up Report Forms (one per patient). Guidelines of reporting AEs to the MUREC IRB shall be followed.

**13.5 Management of serious adverse events**

For all SAEs, the relationship of the events to the study medication shall be determined by the clinicians. If a patient experiences an SAE while receiving treatment for uncomplicated malaria with their assigned study treatment, the study medication will be stopped if the SAE is felt to be probably or definitely related to the study medication and the patient will be treated with quinine. Plans for additional follow-up, including laboratory tests, will be individualized to each subject as appropriate for the clinical setting. If an SAE occurs while a patient is not currently receiving treatment with their study medications, treatment and plans for follow up will be individualized to each subject as appropriate for the clinical setting.

# 14.0 TIMETABLE

# Once all of the appropriate approvals are obtained, a pilot study will begin at one of the study sites and approximately 10 patients will be enrolled over 1 week. Patients enrolled into the pilot study will be followed to study completion but will not have their data included in the final analysis. Enrollment of patients to be included in the analyses will begin the week after the pilot study (assuming no problems arise). Our goal will be to enroll 25-30 patients each week necessitating 5 months to complete the studies at each site. After enrollment is complete at one site, a pilot study will begin at our second study site following the same timeline above. It is anticipated that the second study will also enroll 25-30 patients/week, taking 5 months to complete. Overall it is anticipated that the total time to complete studies at all sites will be 18 - 24 months.

# 15.0 REFERENCES

# APPENDIX 1. PARTICIPANT SELECTION AND ENROLLMENT

Patients attending OPD with history of recent fever

Send to laboratory

Screening thick blood smear

Rapid diagnostic tests (RDTs; results for surveillance purposes only)

Negative smear, or parasitemia Parasitemia ≥ 2,000 or ≤ 200,000

< 2,000 or > 200,000 /ul, and age ≥ 6 months and ≤ 10 years

or age < 6 months or > 10 years

Refer patient to clinician

Refer patient back to OPD

Complete Screening Form

Excluded Passed Initial Screening

Complete Informed Consent Form

Excluded

Refer patient back to OPD

Save screening form only

Assign Study Number

#### Excluded

Complete Case Record Forms

Excluded

Refer back to the Laboratory

Hb < 5.0 gm/dL

Schedule Follow up Visits

Refer to the Study Nurse

Assign Treatment Number

Study thin and thick smear read overnight

Meet additional inclusion criteria on Day 1?

*P. falciparum* monoinfection

Parasite density 2,000-200,000 parasites/ul

**Yes**

**No**

**Excluded on Day 1**

**(Treat as appropriate)**

**Continue with scheduled follow-up outlined in appendix 4.**

# Appendix 2: Patient Screening Form

**STUDY SITE CODE:________ OPD NUMBER: ___________________________**

UMSP SCREENING FORM

| **Names:** | **1. Date: *(dd/mm/yy)*** | **2.Weight (kg):** |
| --- | --- | --- |
| **3. Age*: _______years __________months.** | **4. Gender: M _______ F_______** | |

****Include months only if age < 5 years,if age > 5 years write “X”***

| **SCREENING selection criteria**  ***Patients who are > 6 months-10 years of age and have a positive screening thick blood smear.*** | | |
| --- | --- | --- |
|  | **yes** | **NO** |
| 5. Confirm age > 6 months – 10 years |  |  |
| 6. Fever (> 37.5°C axillary) or history of fever in previous 24 hours |  |  |
| 7. Weight > 5 kg |  |  |
| 8. Ability to participate in 42 day follow-up. |  |  |
|  | **NO** | **YES** |
| 9. Previously participated in this study? |  |  |
| 10. History of serious side effects to study drugs  *If present, indicate drug / side effect:*   Artemether-lumefantrine:________________________   Dihydroartemisinin-piperaquine:______________________   Any other artemisinin compound:_____________________ |  |  |
| 11. Evidence of severe malaria / danger signs  *If “ YES” indicate criteria. If “NO”, leave blank.*   Unarousable coma *(if after convulsion, > 30 min)*   Repeated convulsions *(> 2 within 24 h)*   Recent convulsions *(1-2 within 24 h)*   Altered consciousness*(confusion, delirium,, coma)*   Lethargy   Unable to drink or breast feed   Vomiting everything   Unable to stand/sit due to weakness   Respiratory distress *(labored breathing at rest)*   Jaundice *(yellow coloring of eyes)* |  |  |
| 12. Evidence of concomitant febrile illness  *If “YES”, indicate illness. If “NO”, leave blank.*   Pneumonia/RTI  Measles   Otitis Media  UTI   Gastroenteritis  Other:_________________ |  |  |
|  | **YES** | **NO** |
| 13. Provision of informed consent. |  |  |
| 14. Passed initial screening |  |  |
| 15. If yes assign study number  **Study number U**|___|___|___|___|___| | | |
| 16. Absence of severe anaemia (Hb < 5.0 g/dl) |  |  |
| 17. Absence of persistent vomiting of study medications on day 0 |  |  |
| ***Complete prior to day 1 clinic visit.*** | | |
| 18. *P. falciparum* mono-infection |  |  |
| 19. Parasite density > 2,000/ul |  |  |
| 20. Parasite density < 200,000/ul |  |  |

***If any of the responses fall into the shaded area, exclude the patient from the study***

# APPENDIX 3. UMSP ENROLLMENT FORM.

| **enrollment form (confidential, Not for data entry)** | | |
| --- | --- | --- |
| **1. Study**  **Number:** | **2. OPD**  **Number:** | **3. Start Date:**  ***(dd/mm/yy)*** |

| 4. Patients name: |
| --- |
| 5. Father’s name: |
| 6. Mother’s name: |
| 7. Primary caregiver / guardian’s name and relationship (N/A if mother or father): |
| 8. Sub county of residence. |
| 9. Home parish: |
| 10. LC1/village: |
| 11. Home address and localizing features: |
| 12. a) Phone number: Yes ___No ___  If yes: b) Phone number (s) and the owner(s): |

# APPENDIX 4. Critical steps

**Day 1**

Evaluate patient and complete Case Record Form. Administer study medications (DOT).

**Day 2**

Evaluate patient and complete Case Record Form. Administer study medications (DOT)

Collect thick blood smear and filter paper sample

**Day 3**

Evaluate patient and complete Case Record Form. Collect thick blood smear and filter paper sample

**Days 7, 14, 21**, **28, 35**

Evaluate patient and complete Case Record Form. Collect thick blood smear and filter paper sample

**Any Unscheduled Day (Day 4-41)**

Evaluate patient and complete Case Record Form. If fever, collect thick blood smear with filter paper sample.

**D2 Parasitemia > D 0 parasitemia**

**ETF**

**Treat with oral quinine**

**Severe disease or danger signs**

Do urgent thick smear, FP sample and Hemoglobin

If patient has

1. Temperature > 37.5^0^C with parasitemia

2. Parasite count >25% Day 0 count.

**ETF**

**Treat with oral quinine**

**Day 42**

Evaluate patient and complete Case Record Form. Collect thick blood smear and filter paper sample.

**Severe disease or danger signs**

Do urgent thick smear,

Thin smear

FP sample and Hemoglobin

**Negative smear**

Continue study

Rx at your discretion.

**Negative smear**

Continue study Rx at your discretion.

**Positive blood smear**

ETF

Give/ refer for IV quinine Rx.

**Positive blood smear**

LCF

Give/ refer for IV Quinine Rx.

**ACPR** No parasitemia on day 42 irrespective of fever history or temperature.

**LPF** Parasitaemia day 42 with temperature < 37.5^0^C without being ETF or LCF.

# Day 2 > Day 0

## ETF

Rx with quinine

Refer for care?

# Day 2 < Day 0

Assess for DDx

Cont. study Rx

Refer for care?

# Yes

## ETF

Rx with quinine

'

If patient has

1. Temp > 37.5°C with parasitemia

2. History of fever in the last 24 hours with parasitemia.

Do Hemoglobin and thin blood smear

LCF

Treat with oral quinine

Patients who are LPF’s should be treated with oral quinine.

# Appendix 5. Informed Consent

**Study number:__________________________**

**RESEARCH PARTICIPANT INFORMED CONSENT FORM**

**Protocol Title:** Comparison of artemether-lumefantrine and dihydroartemisinin-piperaquine for treatment of uncomplicated malaria in Uganda: evaluation of efficacy, tolerability, and safety

**Site of Research:** UMSP Sentinel Sites, Uganda

**Sponsor:** Centers for Disease Control.

**Ugandan Principal Investigator**: Fred Wabwire-Mangen, MBChB, DTM&H, MPH, PhD

**U.S. Principal Investigator:** Grant Dorsey, MD, PhD

**Date:** 11 December 2005

**PURPOSE OF THE STUDY**

This research study is being done to learn more about the treatment of malaria. We would like to know what the best and safest treatment is for malaria in Uganda. To do this, we are carrying out a research study to compare different combinations of malaria drugs. About 400 participants will be involved in this study. The study is conducted by the Uganda Malaria Surveillance Project (UMSP), which is a collaboration between the Ugandan Ministry of Health and academic partners from Uganda and the USA.

**HOW THE STUDY IS DONE**

The child under your case has been diagnosed with malaria. If you agree to allow your child to participate in this study, the child under your care will be treated with artemether-lumefantrine (Coartem) or dihydroartemisinin-piperaquine (Duo-Cotecxin), two new treatments for malaria. After the treatment, your child will be followed for 42 days to see if the malaria infection is cured. If your child does not get better after treatment, he or she will be given additional medication. The treatment that your child will receive will be determined by a process of randomization. Randomization means that the treatment given to your child will be based on chance, similar to pulling a number out of a hat. The chance of being placed into each of the treatment groups is the same. You will not be told which treatment your child has been assigned to receive. You are being asked to allow your child to participate in this study for up to 42 days or until such a time as you or the study doctors decide that your child should no longer participate in the study. The study may be discontinued by the sponsor at any time, and for any reason. The study doctors may withdraw your child from the study for the following reasons:

1. If your child should receive malaria medicines not prescribed by the study doctors
2. If your child develops an illness in addition to malaria which makes it difficult for the doctors to tell which problem is causing fever
3. If you chose to withdraw your consent to have your child participate in the study
4. If we are unable to locate your child for both doses of study medications on day 0-2, within 24 hours on day 3, or within 48 hours on days 4-42 of the study follow-up period.

PROCEDURES

- 1. The study doctors will examine your child today.
  2. A blood sample will be collected. Approximately 6 drops of blood will be taken by fingerprick to examine for malaria parasites, to measure the blood count, and to store blood samples on paper for future laboratory tests that will not impact on the health care of your child.
  3. If the diagnosis of malaria is confirmed, and your child is eligible for the study, treatment with artemether-lumefantrine (Coartem) or dihydoartemisinin-piperaquine (Duo-Cotexin) will be given. Artemether-lumefantrine is the government’s new recommended first-line treatment for malaria and dihydroartemininin-piperquine is a new antimalarial drug being considered by the government for future wide-spread use. Both treatments being evaluated in this study are given as pills twice a day for three days. If your child is too young or unable to swallow pills, the pills will be crushed and added to sugar and water.
  4. You will be asked to return to the clinic at least 9 more times over the next 6 weeks so that the success of the treatment can be judged. At each of the follow-up visits, your child will be examined by the study doctors and, at 8 of these visits, approximately 6 drops of blood will be taken by fingerprick to examine for malaria parasites and to save on paper. The blood counts will be measured again on the last day.
  5. If your child misses an appointment, the home health visitor will visit you at your home to find out why you missed the appointment and bring your child to the clinic for assessment.
  6. If, at any time, the treatment given to your child does not seem to be working well, it will be changed to quinine. Quinine is the government recommended therapy for patients who fail treatment for malaria. Your child may develop malaria that is severe even after receiving treatment with study medications. If your child shows any evidence of severe malaria (including persistent vomiting, low blood counts, shaking or fits, confusion, or you cannot wake your child) your child will be referred for possible admission to hospital.
  7. There will be someone at the study clinic every day from 8:00 am to 5:00 pm and at night. Your child can be brought to the clinic and will be seen by a doctor anytime they are ill during the next 42 days.

**RISKS AND DISCOMFORTS**

1. Randomization: Which treatment your child is given will be determined by chance. The treatment your child receives may prove to be less effective or have more side effects than the other study treatment or than other available treatments. This will not be known until after the study is completed.
2. Medication side effects: Serious health problems have rarely been reported following treatment with the study medications. Artemether-lumefantrine (Coartem) - sleep problems, headache, dizziness, a feeling of a rapid or irregular heart beat, diarrhea, vomiting, pain in the abdomen, rash, itching, cough, muscle and joint aches, and fatigue may occur occasionally (1-10% of the time) in patients. Dihydoartemisinin-piperaquine (Duo-Cotexin)- nausea, diarrhea, vomiting, abdominal pain, anorexia, itching, rashes, and dizziness may occur occasionally (1-10% of the time) in patients. Your child will be monitored closely after receiving treatment for malaria with the study medications for any possible side effects of the drugs and will receive appropriate medical care for any such problem during the course of the study. If your child develops severe shaking or fits, is having trouble breathing, cannot eat, drink, or breast feed, or cannot be woken from sleep, they should be brought to the clinic as soon is possible.
3. Blood draws: The risks of drawing blood from a fingerprick include temporary discomfort, bruising, skin infection, and fainting. The amount of blood removed will be too small to affect your child’s health.
4. Unknown Risks: The research treatments may have side effects that no one knows about yet. The researchers will let you know if they learn anything that might make you change your mind about your child’s participation in the study.
5. Confidentiality: Participation in research may involve a loss of privacy, but information about your child will be handled as confidentially as possible. Under certain conditions, people responsible for making sure that the research is done properly may review your child’s study records. This might include people involved with sponsoring or monitoring the study. All of these people are also required to keep your child’s identity confidential. Otherwise, the information that identifies your child will not be given out to people who are not working on the study.

**BENEFITS**

1. The potential benefit to your child is that the treatment received may prove to be more effective than the other study treatments or than other available treatments, although this cannot be guaranteed.
2. Your child will receive clinical care from the medical officers and nurses of the project staff in the study clinic. This will include care for unscheduled sick visits.
3. The knowledge gained from this study will help the country of Uganda in determining the best treatment for uncomplicated malaria.

**COST/PAYMENT**

After enrolment in the study, you will not be charged for clinic visits or treatment. You (or your child) will not be paid for participation in the study. You will be reimbursed for transport costs to and from the clinic for any visit that your child requires.

**ALTERNATIVES TO PARTICIPATION**

Your child’s participation in this study is completely voluntary. If you decide you do not want to participate in the study, your child will be treated with the current standard of care in Uganda. The current standard of care of the treatment of uncomplicated malaria in Uganda is chloroquine plus sulfadoxine-pyrimethamine, although the government is in the process of changing the recommended therapy to artemether-lumefantrine. If you decide to withdraw your child from the study at any time and for any reason, this will not affect your child’s care at the outpatient department, where standard care for all medical problems is available. During the study, you will be informed promptly of any new information that may influence your willingness to continue participation in the study.

**WITHDRAWAL**

Should you or your study doctors decide to withdraw your child from the study, your child will still be eligible for care according to the standard clinic procedures.

**USE OF THE RESULTS**

The findings from this study may be published in a medical journal. The study participants will not be identified by name. After the study is completed, you may request an explanation of the study results.

**TREATMENT AND COMPENSATION FOR INJURY**

If your child is injured or you have questions about injuries as a result your child being in the study, please contact the doctors in the study clinic. The services at the public health facility will be open to you in case of any such injury. However, neither the Uganda Malaria Surveillance Project nor the sponsors of this study have a program to cover your costs if your child is hurt or has other bad results.

**QUESTIONS**

This study has been explained to you by the person who signed below and your questions were answered. If you have any other questions about the study, you may call Dr. Katabira (041-530020), chairman of the Makerere University Research and Ethic Committee.

**JOINING OF YOUR OWN FREE WILL**

PARTICIPATION IN RESEARCH IS VOLUNTARY. You have the right to refuse to allow your child to participate or to withdraw at any point in this study without penalty or loss of benefits to which you are otherwise entitled.

**WHAT YOUR SIGNATURE OR THUMBPRINT MEANS**

Your signature or thumbprint below means that you understand the information given to you about your child’s participation in the study and in this consent form. If you wish for your child to participate in this study, you should sign or place your thumbprint below. You will also be asked to sign another informed consent forms for the use of stored specimens.

Name of Participant (printed)

Name of Parent/Guardian

Signature or Fingerprint * of Parent/Guardian Date/Time

*If the parent or guardian is unable to read and/or write, an impartial witness should be present during the informed consent discussion. After the written informed consent form is read and explained to the parent or guardian, and after they have orally consented to their child’s participation in the trial, and have either signed the consent form or provided their fingerprint, the witness should sign and personally date the consent form. By signing the consent form, the witness attests that the information in the consent form and any other written information was accurately explained to, and apparently understood by, the parent or guardian, and that informed consent was freely given by the parent or guardian.

Name of Person Witnessing Consent (printed)

Signature of Person Witnessing Consent Date/Time

**Study number**:_______________________

**INFORMED CONSENT FOR FUTURE USE OF BIOLOGICAL SPECIMENS**

**Protocol Title:** Comparison of artemether-lumefantrine and dihydroartemisinin-piperaquine for treatment of uncomplicated malaria in Uganda: evaluation of efficacy, tolerability, and safety

**Site of Research:** UMSP Sentinel Sites, Uganda

**Sponsor:** Centers for Disease Control.

**Ugandan Principal Investigator**: Fred Wabwire-Mangen, MBChB, DTM&H, MPH, PhD

**U.S. Principal Investigator:** Grant Dorsey, MD, PhD

**Date:** 11 December 2005

**INTRODUCTION**

While your child is in this study, blood samples may be taken that may be useful for future research. These samples will be stored long-term at Makerere University Medical School and the University of California, San Francisco. Samples may also be shared with investigators at other institutions.

**WHAT SAMPLES WILL BE USED FOR**

Your child’s blood and the malaria parasites in it will be used to study malaria and the response of this disease to treatment. Results of these studies will not affect your child's care.

1. These samples will be used for future research to learn more about malaria and other diseases.
2. Your child’s samples will be used only for research and will not be sold or used for the production of commercial products.
3. Genetic research may be performed on samples. However, no genetic information obtained from this research will be placed in your child’s medical records. These samples will be identified only by codes so that they cannot be readily identified with your child.
4. For any future genetic studies done on your child’s samples not related to the current study, permission shat first be sought from the Makerere University Research and Ethics Committee Institutional Review Board and the University of California, San Francisco Committee on Human Research.

**LEVEL OF IDENTIFICATION**

Your child’s samples will be coded so that the child’s name cannot be readily identified. Reports about research done with the samples will not be put in the medical record and will be kept confidential to the best of our ability.

In the future, researchers studying your child’s samples may need to know more about your child, such as information about age and gender. If this information is already available because of your child’s participation in a study, it may be provided to the researcher. Your child’s name or anything that might identify you/them personally will not be provided. You will not be asked to provide additional consent.

**RISKS**

There are few risks to your child from future use of the samples. A potential risk might be the release of information from your child’s health or study records. Reports about research done with your child’s samples will not be put in the health record, but will be kept with the study records. The study records will be kept confidential as far as possible.

**BENEFITS**

There will be no direct benefit to your child. From studying your child’s samples we may learn more about malaria or other diseases: how to prevent them, how to treat them, how to cure them.

**RESEARCH RESULTS/MEDICAL RECORDS**

##

1. Results from future research using your child’s samples may be presented in publications and meetings but patient names will not be identified.
2. Reports from future research done with your child’s samples will not be given to you or the doctor. These reports will not be put in your child’s medical record.

**QUESTIONS**

The future use of your child’s specimens has been explained to you by the person who signed below and your questions were answered. If you have any other questions about the information here, you may call Dr. Katabira (041-530020), chairman of the Makerere University Research and Ethic Committee.

**FREEDOM TO REFUSE**

You can change your mind at any time about allowing your child’s samples to be used for future research. If you do, contact Dr. Adoke Yeka Yeka (077473533) or Dr. Hasifa Bukirwa (071562083) at the Ugandan Malaria Surveillance Project offices. Then your child’s samples will no longer be made available for research and will be destroyed. Whether or not you allow us to use your child’s samples in future research will not have any effect on your child’s participation in this study or future participation in other studies.

**WHAT YOUR SIGNATURE OR THUMBPRINT MEANS**

Your signature or thumbprint below means that you understand the information given to you in this consent form about your child’s specimens and cultures to be used for future research. If you wish to allow your child’s specimens and cultures to be used for future research, you should sign or thumbprint below.

Name of Participant (printed)

Name of Parent/Guardian

Signature or Fingerprint * of Parent/Guardian Date/Time

*If the parent or guardian is unable to read and/or write, an impartial witness should be present during the informed consent discussion. After the written informed consent form is read and explained to the parent or guardian, and after they have orally consented to their child’s participation in the trial, and have either signed the consent form or provided their fingerprint, the witness should sign and personally date the consent form. By signing the consent form, the witness attests that the information in the consent form and any other written information was accurately explained to, and apparently understood by parent or guardian, and that informed consent was freely given by the parent or guardian.

Name of Person Witnessing Consent (printed)

Signature of Person Witnessing Consent Date/Time

# Appendix 6a. Weight-based administration of study medications for DP group

| **Weight (kg)** | **Dihydroartemisinin-piperaquine (DP 40mg/320mg)** | | | | | **Placebo tablets** | | |
| --- | --- | --- | --- | --- | --- | --- | --- | --- |
|  | **Day 0** | **Day 1** | **Day 2** | **Total DHA dose**  **(mg/kg)** | **Total PQ dose**  **(mg/kg)** | **Day 0** | **Day 1** | **Day2** |
|  | **am** | **am** | **am** |  |  | **pm** | **pm** | **pm** |
| 5 | ¼ | ¼ | ¼ | 6.0 | 48.0 | ¼ | ¼ | ¼ |
| 6 | ½ | ½ | ½ | 10 | 80.0 | ½ | ½ | ½ |
| 7 | ½ | ½ | ½ | 8.6 | 68.6 | ½ | ½ | ½ |
| 8 | ½ | ½ | ½ | 7.5 | 60.0 | ½ | ½ | ½ |
| 9 | ½ | ½ | ½ | 6.7 | 53.5 | ½ | ½ | ½ |
| 10 | ½ | ½ | ½ | 6.0 | 48.0 | ½ | ½ | ½ |
| 11 | ¾ | ¾ | ¾ | 8.2 | 65.5 | ¾ | ¾ | ¾ |
| 12 | ¾ | ¾ | ¾ | 7.5 | 60.0 | ¾ | ¾ | ¾ |
| 13 | ¾ | ¾ | ¾ | 6.9 | 55.4 | ¾ | ¾ | ¾ |
| 14 | ¾ | ¾ | ¾ | 6.4 | 51.4 | ¾ | ¾ | ¾ |
| 15 | 1 | 1 | 1 | 8.0 | 64.0 | 1 | 1 | 1 |
| 16 | 1 | 1 | 1 | 7.5 | 60.0 | 1 | 1 | 1 |
| 17 | 1 | 1 | 1 | 7.1 | 56.5 | 1 | 1 | 1 |
| 18 | 1 | 1 | 1 | 6.7 | 53.3 | 1 | 1 | 1 |
| 19 | 1 | 1 | 1 | 6.3 | 50.5 | 1 | 1 | 1 |
| 20 | 1 ¼ | 1 ¼ | 1 ¼ | 7.5 | 60.0 | 1 ¼ | 1 ¼ | 1 ¼ |
| 21 | 1 ¼ | 1 ¼ | 1 ¼ | 7.1 | 57.1 | 1 ¼ | 1 ¼ | 1 ¼ |
| 22 | 1 ¼ | 1 ¼ | 1 ¼ | 6.8 | 54.5 | 1 ¼ | 1 ¼ | 1 ¼ |
| 23 | 1 ¼ | 1 ¼ | 1 ¼ | 6.5 | 52.3 | 1 ¼ | 1 ¼ | 1 ¼ |
| 24 | 1 ½ | 1 ½ | 1 ½ | 7.5 | 60.0 | 1 ½ | 1 ½ | 1 ½ |
| 25 | 1 ½ | 1 ½ | 1 ½ | 7.2 | 57.6 | 1 ½ | 1 ½ | 1 ½ |
| 26 | 1 ½ | 1 ½ | 1 ½ | 6.9 | 55.4 | 1 ½ | 1 ½ | 1 ½ |
| 27 | 1 ½ | 1 ½ | 1 ½ | 6.7 | 53.3 | 1 ½ | 1 ½ | 1 ½ |
| 28 | 1 ½ | 1 ½ | 1 ½ | 6.4 | 51.4 | 1 ½ | 1 ½ | 1 ½ |
| 29 | 1 ¾ | 1 ¾ | 1 ¾ | 7.2 | 57.9 | 1 ¾ | 1 ¾ | 1 ¾ |
| 30 | 1 ¾ | 1 ¾ | 1 ¾ | 7.0 | 56.0 | 1 ¾ | 1 ¾ | 1 ¾ |
| 31 | 1 ¾ | 1 ¾ | 1 ¾ | 6.8 | 54.2 | 1 ¾ | 1 ¾ | 1 ¾ |
| 32 | 1 ¾ | 1 ¾ | 1 ¾ | 6.6 | 52.3 | 1 ¾ | 1 ¾ | 1 ¾ |
| 33 | 1 ¾ | 1 ¾ | 1 ¾ | 6.4 | 50.9 | 1 ¾ | 1 ¾ | 1 ¾ |
| 34 | 2 | 2 | 2 | 7.1 | 56.5 | 2 | 2 | 2 |
| 35 | 2 | 2 | 2 | 6.9 | 54.9 | 2 | 2 | 2 |
| 36 | 2 | 2 | 2 | 6.7 | 53.3 | 2 | 2 | 2 |
| 37 | 2 | 2 | 2 | 6.5 | 51.2 | 2 | 2 | 2 |
| 38 | 2 | 2 | 2 | 6.3 | 50.5 | 2 | 2 | 2 |
| 39 | 2 | 2 | 2 | 6.2 | 49.2 | 2 | 2 | 2 |
| 40 | 2 | 2 | 2 | 6.0 | 48.0 | 2 | 2 | 2 |

# Appendix 6b. Weight-based administration of study medications for AL group

| **Weight (kg)** | **Artemether-lumefantrine (AL 20mg/120mg)** | | | | | |
| --- | --- | --- | --- | --- | --- | --- |
|  | **Day 0** | | **Day 1** | | **Day 2** | |
|  | am | pm | am | pm | am | pm |
| 5 | 1 | 1 | 1 | 1 | 1 | 1 |
| 6 | 1 | 1 | 1 | 1 | 1 | 1 |
| 7 | 1 | 1 | 1 | 1 | 1 | 1 |
| 8 | 1 | 1 | 1 | 1 | 1 | 1 |
| 9 | 1 | 1 | 1 | 1 | 1 | 1 |
| 10 | 1 | 1 | 1 | 1 | 1 | 1 |
| 11 | 1 | 1 | 1 | 1 | 1 | 1 |
| 12 | 1 | 1 | 1 | 1 | 1 | 1 |
| 13 | 1 | 1 | 1 | 1 | 1 | 1 |
| 14 | 1 | 1 | 1 | 1 | 1 | 1 |
| 15 | 2 | 2 | 2 | 2 | 2 | 2 |
| 16 | 2 | 2 | 2 | 2 | 2 | 2 |
| 17 | 2 | 2 | 2 | 2 | 2 | 2 |
| 18 | 2 | 2 | 2 | 2 | 2 | 2 |
| 19 | 2 | 2 | 2 | 2 | 2 | 2 |
| 20 | 2 | 2 | 2 | 2 | 2 | 2 |
| 21 | 2 | 2 | 2 | 2 | 2 | 2 |
| 22 | 2 | 2 | 2 | 2 | 2 | 2 |
| 23 | 2 | 2 | 2 | 2 | 2 | 2 |
| 24 | 2 | 2 | 2 | 2 | 2 | 2 |
| 25 | 3 | 3 | 3 | 3 | 3 | 3 |
| 26 | 3 | 3 | 3 | 3 | 3 | 3 |
| 27 | 3 | 3 | 3 | 3 | 3 | 3 |
| 28 | 3 | 3 | 3 | 3 | 3 | 3 |
| 29 | 3 | 3 | 3 | 3 | 3 | 3 |
| 30 | 3 | 3 | 3 | 3 | 3 | 3 |
| 31 | 3 | 3 | 3 | 3 | 3 | 3 |
| 32 | 3 | 3 | 3 | 3 | 3 | 3 |
| 33 | 3 | 3 | 3 | 3 | 3 | 3 |
| 34 | 3 | 3 | 3 | 3 | 3 | 3 |
| 35 | 4 | 4 | 4 | 4 | 4 | 4 |
| 36 | 4 | 4 | 4 | 4 | 4 | 4 |
| 37 | 4 | 4 | 4 | 4 | 4 | 4 |
| 38 | 4 | 4 | 4 | 4 | 4 | 4 |
| 39 | 4 | 4 | 4 | 4 | 4 | 4 |
| 40 | 4 | 4 | 4 | 4 | 4 | 4 |

# APPENDIX 7. CLASSIFICATION OF RESPONSE TO TREATMENT

**Early Treatment Failure (ETF)**

| **ETF**   - Development of danger signs or severe malaria on Days 0-3 in the presence of parasitemia - Parasitemia on day 2 higher than Day 0 count irrespective of axillary temperature. - Parasitemia on Day 3 with axillary temperature > 37.5 ^0^C - Parasitemia on Day 3 > 25% of count on Day 0. - Severe AE requiring change in treatment Days 0-2 (before last dose of study drug given) |
| --- |

**Late Treatment Failure (LTF)**

| **Late Clinical Failure (LCF):**   - Development of danger signs or severe malaria Days 4-42 in the presence of parasitemia, without previously meeting any of the criteria of early treatment failure - Presence of parasitemia and axillary temperature > 37.5°C, (or history of fever in past 24 hours), on any day from Day 4 to Day 42, without previously meeting any of the criteria of early treatment failure   **Late Parasitological Failure (LPF):**   - Presence of parasitemia on Day 42 and axillary temperature < 37.5°C, without previously meeting any of the criteria of early treatment failure or late clinical failure |
| --- |

Adequate Clinical and Parasitological Response (ACPR)

| **ACPR**   - Absence of parasitemia on Day 42 irrespective of temperature without previously meeting any of the criteria of early treatment failure or late clinical failure or late parasitological failure. |
| --- |

# Appendix 8. Criteria for Severe Malaria/Danger Signs

**Severe Malaria**

- Unarousable coma *(if after convulsion, > 30 min)*
- Repeated convulsions *(> 2 within 24 h)*
  - Severe anemia *(Hb < 5.0 g/dL)*
  - Respiratory distress *(labored breathing at rest)*
  - Jaundice *(yellow coloring of eyes)*

**Danger Signs**

- Recent convulsions *(1-2 within 24 h)*
- Altered consciousness  *(confusion, delirium, psychosis)*
- Lethargy
  - Unable to drink or breast feed
  - Vomiting everything
  - Unable to stand/sit due to weakness

# Appendix 9. Guidelines for Grading Patient Symptoms, signs and laboratory findings.

## Table A. Guidelines for grading patient symptoms.

|  | **Grade 1**  **MILD** | **Grade 2**  **MODERATE** | **Grade 3**  **SEVERE** | **Grade 4**  **LIFE THREATENING** |
| --- | --- | --- | --- | --- |
| **Subjective fever in the past 24 h** | N/A | Present (Yes) | N/A | N/A |
| **Weakness** | Mild decrease in activity; For children – weak, but still playing | Moderate decrease in activity; For children – weak, and playing limited | Not participating in usual activities; For children – not playing | Prostration |
| **Muscle and/or joint aches*** | Mild and/or localized complaints | Diffuse complaints | Objective weakness; function limited | N/A |
| **Headache*** | Mild, no treatment required | Transient, moderate; treatment required | Severe, constant; requires narcotic therapy | Intractable; requires repeated narcotic therapy |
| **Anorexia** | Decreased appetite, but still taking solid food | Decreased appetite, avoiding solid food but taking liquids | Appetite very decreased; Refusing to breast feed, no solids or liquids taken (< 2 years < 12 hr; > 2 years < 24 hr) | Appetite very decreased; Refusing to breast feed, no solids or liquids taken (< 2 years > 12 hr; > 2 years > 24 hr) |
| **Nausea*** | Mild, transient feeling of impending vomiting; maintains reasonable intake | Moderate and/or constant feeling of impending vomiting; intake decreased | Severe, constant feeling of impending emesis; intake decreased significantly | N/A |
| **Vomiting** | 1 episode per day | 2-3 episodes per day | Orthostatic hypotension or IV fluids required | Hypotensive shock or 46nrolment46ation required for IV fluid therapy |
| **Abdominal pain*** | Mild (1-3 on a scale of 1 to 10) | Moderate (4-6 on a scale of 1 to 10) | Moderate to severe (> 7 on a scale of 1 to 10) | Severe – 46nrolment46at for treatment |
| **Diarrhea** | Transient 3-4 loose stools/day | 5-7 loose stools/day | Orthostatic hypotension or > 7 loose stools/day or IV fluids required | Hypotensive shock or 46nrolment46ation for IV fluid therapy required |
| **Cough** | Transient / intermittent | Persistent / constant | Uncontrolled | Cyanosis, stridor, severe shortness of breath |
| **Pruritis** | Transient pruritis | Pruritis that disturbs sleep | Severe, constant pruritis, sleep disturbed | N/A |
| **Tinnitus*** | Mild, transient ringing or roaring sound | Moderate, persistent ringing or roaring sound | Severe ringing or roaring sound with associated hearing loss | N/A |
| **Behavioural changes** | Mild difficulty concentrating; mild confusion or agitation; activities of daily living unaffected; no treatment | Moderate confusion or agitation; some limitation of activities of daily living; minimal treatment | Severe confusion or agitation; Needs assistance for activities of daily living; therapy required | Toxic psychosis; 46nrolment46ation required |
| **“Flu”**  **(viral URI)** | Mild nasal congestion, mild rhinorrhea | Moderate nasal congestion, moderate rhinorrhea | N/A | N/A |
| **Allergic reaction** | N/A | N/A | Urticaria | Severe urticaria  anaphylaxis, angioedema |
| **Convulsion** | N/A | N/A | Localized or generalized seizure | Status epilepticus |
| *** Assess only in children > 3 years of age. Answer N/A for younger children and those unable to answer.** | | | | |

Reference – Based on WHO Toxicity Grading Scale for Determining the Severity of Adverse Events

## Table B. Guidelines for Physical Examination

| Dehydration | Assess skin touch and turgor, mucous membranes, eyes, crying, fontanelle, pulse, urine output |
| --- | --- |
| **Jaundice** | Assess for yellowing of the sclera. Also evaluate the palpepral conjunctiva, lips, and skin. |
| **Chest** | Observe the rate, rhythm, depth, and effort of breathing. Check the patient’s colour for cyanosis.  The maximum acceptable respiratory rate by age: < 2 months = 60, 2-12 months = 50, 1-5 years = 40, above 5 years = 30.  Inspect the neck for the position of the trachea, for supraclavicular retractions, and for contraction of the sternomastoid or other accessory muscles during inspiration.  Auscultate the anterior and posterior chest for normal breath sounds and any adventitious sounds (crackles or rales, wheezes, and rhonchi). *Crackles are intermittent, non-musical, fine or coarse sounds that may be due to abnormalities of the lungs (pneumonia, fibrosis, early congestive heart failure) or airways (bronchitis or bronchiectasis). Wheezes are high-pitched and result from narrowed airways. Rhonchi are relatively low-pitched and suggest secretions in large airways.*  If abnormalities are identified, evaluate for transmitted voice sounds. In addition, palpate the chest to assess for tactile fremitus, and percuss the chest to assess for areas of dullness*. Normal, air-filled lungs emit predominantly vesicular breath sounds, transmit voice sounds poorly with “ee” = “ee”, and have no tactile fremitus. Airless lung, as in lobar pneumonia, emits bronchial breath sounds, transmits spoken words clearly with “ee” = “aay” (egophany), and has an increase in tactile fremitus.* |
| **Abdomen** | Inspect and ausculate the abdomen. Listen for bowel sounds in the abdomen before palpating it. Palpate the abdomen in all 4 quadrants lightly and then deeply. Assess the size of the liver and spleen. To assess for peritoneal inflammation, look for localised and rebound tenderness, and voluntary or involuntary rigidity. |
| **Skin** | Inspect the skin for colour, turgor, moisture, and lesions. If lesions are present, note their location and distribution (diffuse or localised), arrangement (linear, clustered, annular, dermatomal), type (macules, papules, vesicles) and colour. |
| **Tablet test** | For children > 9 months of age, ask the patient to pick a tablet (or equivalent object) up off a flat surface using the thumb and index finger of their dominant hand*. This tests for co-ordination of the upper extremity assessing the function of the motor system, cerebellar system, vestibular system (for coordinating eye and body movements) and the sensory system, for position sense. When testing small children, be aware that they will likely attempt to put the object into their mouth.* |

## Table C. Grading Physical Examination Findings

|  | **Grade 1**  **MILD** | **Grade 2**  **MODERATE** | **Grade 3**  **SEVERE** | **Grade 4**  **LIFE-THREATENING** |
| --- | --- | --- | --- | --- |
| **Temperature* (axillary)** | 37.5-37.9°C | 38.0-39.5°C | > 39.5°C | Sustained fever, equal or greater than 40.0°C for longer than 5 days |
| **Dehydration** | Less than 2 of the following:  Restless, irritable  Sunken eyes  Drinks eagerly, thirsty  Skin pinch goes back slowly | 2 of the following:  Restless, irritable  Sunken eyes  Drinks eagerly, thirsty  Skin pinch goes back slowly | Two of the following:  Lethargic or unconscious  Sunken eyes  Not able to drink or drinking poorly  Skin pinch goes back very poorly | Two of the following + shock:  Lethargic or unconscious  Sunken eyes  Not able to drink or drinking poorly  Skin pinch goes back very poorly |
| **Jaundice** | Slight yellowing of sclera and conjunctiva | Moderate yellowing of sclera and conjunctiva, yellowing of mucous membranes | Severe yellowing of sclera and conjunctiva, yellowing of skin | N/A |
| **Chest** | Mildly increased RR (for age, temperature), transient or localised adventitious sounds | Moderately increased RR, diffuse or persistent adventitious sounds | Rapid RR (< 2 months > 60, 2-12 months > 50, 1-5 years > 40, adults > 30)* nasal flaring, retractions | Cyanosis |
| **Abdomen** | Normal bowel sounds, mild localised tenderness, and/or liver palpable 2-4 cm below the right costal margin (RCM), and/or spleen palpable, and/or umbilical hernia present | Normal or mildly abnormal bowel sounds, moderate or diffuse tenderness; and/or mild to moderately enlarged liver (4-6 cm below the RCM) and/or spleen palpable up to half-way between umbilicus and symphysis pubis | Severely abnormal bowel sounds, severe tenderness to palpation. Evidence of peritoneal irritation and/or significant enlargement of liver (> 6 cm below the RCM) and/or spleen palpable beyond half-way between umbilicus and symphysis pubis | Absent bowel sounds. Involuntary rigidity |
| **Skin†** | Localised rash, erythema, or pruritis | Diffuse, maculopapular rash, dry desquamation | Vesiculation, moist desquamation, or ulceration | Exfoliative dermatitis, mucous membrane involvement or erythema multiforme or suspected Stevens-Johnson or necrosis requiring surgery |

|  | **Grade 1**  **MILD** | **Grade 2**  **MODERATE** | **Grade 3**  **SEVERE** | **Grade 4**  **LIFE-THREATENING** |
| --- | --- | --- | --- | --- |
| **Hearing** | *< 4 years: N/A*  > 4 years: Decreased hearing in one ear | *< 4 years: N/A*  > 4 years: Decreased hearing in both ears or severe impairment in one ear | *< 4 years: Any evidence of hearing impairment*  > 4 years: Severe impairment in both ears | N/A |
| **Tablet test** | Difficulty grasping tablet but able to pick up | Unable to pick up tablet without dropping | Unable to grasp tablet | N/A |
| **Clinical symptoms / sign *(not otherwise specified)*** | No treatment required; monitor condition | Treatment required | Requires treatment and possible hospitalisation | Requires active medical intervention, hospitalisation, or hospice care |

- Reference – The Harriet Lane Handbook, 15^th^ edition, 2000

† Reference – WHO Toxicity Grading Scale for Determining the Severity of Adverse Events

## TABLE D. Guidelines for Grading of Laboratory Abnormalities

|  | **Grade 1**  **MILD** | **Grade 2**  **MODERATE** | **Grade 3**  **SEVERE** | **Grade 4**  **LIFE-THREATENING** |
| --- | --- | --- | --- | --- |
| **Haemoglobin**  ***(****g/dL)* | 9.0 – 9.9 | 7.0 – 8.9 | 5.0 – 6.9 | < 5.0 |

Reference – The Harriet Lane Handbook, 15^th^ edition, 2000†

Reference – WHO Toxicity Grading Scale for Determining the Severity of Adverse Events

# Appendix 10. Adverse Event - Follow-up Report

| **ADVERSE EVENT FORM – FOLLOW-UP REPORT** | | |
| --- | --- | --- |
| **1. Study**  **Number: U**|___|___|___|___|___| | **2. Day 0 Date:** |___|___|/|___|___|/|___|___|  ***day month year*** | **3. OPD**  **Number: ____________**| |

| Date of follow-up: |___|___|/|___|___|/|___|___|  *day month year* | | | Study Day: | Temp: | |
| --- | --- | --- | --- | --- | --- |
| Progress Note:  _______________________________________________________  _______________________________________________________  _______________________________________________________  _______________________________________________________  _______________________________________________________  _______________________________________________________  _______________________________________________________  _______________________________________________________  _______________________________________________________  _______________________________________________________  _______________________________________________________  _______________________________________________________  _______________________________________________________  _______________________________________________________  _______________________________________________________  _______________________________________________________  _______________________________________________________ | | | Laboratory results / Other comments: | | |
| Date of follow-up: |___|___|/|___|___|/|___|___|  *day month year* | | | Study Day: | | Temp: |
| Progress Note:  _______________________________________________________  _______________________________________________________  _______________________________________________________  _______________________________________________________  _______________________________________________________  _______________________________________________________  _______________________________________________________  _______________________________________________________  _______________________________________________________  _______________________________________________________  _______________________________________________________  _______________________________________________________  _______________________________________________________  _______________________________________________________  _______________________________________________________  _______________________________________________________  _______________________________________________________ | | | Laboratory results / Other comments: | | |
| Outcome:   Resolved   Ongoing   Died | If resolved, date of resolution:  |___|___|/|___|___|/|___|___|___|___|  *day month year* | Investigator’s signature:  ________________________________  Date:________________________________ | | | |

# Appendix 11. Serious Adverse Event Form – Initial report.

| **SERIOUS adverse even form – initial report** | | |
| --- | --- | --- |
| **1. Study**  **Number: U**|___|___|___|___|___| | **2. Day 0 Date:** |___|___|/|___|___|/|___|___|  ***day month year*** | **3. OPD**  **Number:**  __________ |

| Event description:______________________________________________________________________________  *(symptom, sign, or laboratory abnormality)* | | | | |
| --- | --- | --- | --- | --- |
| Date of event onset:  |___|___|/|___|___|/|___|___|___|___|  day month year | | Date event reported:  |___|___|/|___|___|/|___|___|___|___|  day month year | | Indicate reason for serious AE:   Fatal   Life-threatening   Resulted in significant /  persistent disability or  incapacity   Resulted in hospitalization   Prolonged hospitalization   Required medical / surgical  intervention to prevent serious  outcome   Other:____________________ |
| Maximum event severity:   Moderate   Severe   Life-threatening | | Maximum relationship to study drugs:   None   Unlikely   Possible   Probable   Definite | |  |
| Was the event unexpected?  Yes ____No____ | |  |  |  |
| Clinical history:  ________________________________________  ________________________________________  ________________________________________  ________________________________________  ________________________________________  ________________________________________  ________________________________________  ________________________________________  ________________________________________  ________________________________________  ________________________________________  ________________________________________  ________________________________________  ________________________________________  ________________________________________  ________________________________________  ________________________________________  ________________________________________  ________________________________________  ________________________________________  ________________________________________  ________________________________________ | | | Relevant past medical history:  ________________________________________  ________________________________________  ________________________________________  ________________________________________  ________________________________________ | |
|  |  |  | Concomitant medications:  1.__________________________________________  2.__________________________________________  3.__________________________________________  4.__________________________________________  5.__________________________________________ | |
|  |  |  | Action taken: (tick all that apply)   No change in current management   Study medication discontinued   Specific treatment given   Patient hospitalized   Laboratory tests obtained   Other:____________________   Other:____________________ | |
| Date form completed:  |___|___|/|___|___|/|___|___|___|___|  *day month year* | Investigator’s name (printed): ________________________________  Investigator’s signature: ______________________________________ | | | |

# Appendix 12. Clinical Record forms

| **UMSP clinical record form (1):** | | | | | | | | |
| --- | --- | --- | --- | --- | --- | --- | --- | --- |
| **Patient**  **Initials:** | **1. Study Number: U**|___|___|___|___|___| | | **2. Day 0 Date:** |___|___|/|___|___|/|___|___|  ***day month year*** | | | | **3. OPD Number:** _____________ | |
| **4. Age:_______years______months**  *(include months only if age < 5 years, else write “X”)* | | **5. Gender: M _____F _____** | | | **6. Weight** *(kg):* | **7. Known drug allergies: Yes ___No ___Unknown ___**  **If yes, describe___________________________________** | | |
| ***List all medications taken within the last 2 weeks*** | | | | | | | | |
| **Drug** *(if name unknown, list by letter – “Unknown Drug A”)* **(a)** | | | | **Dose (b)** | | | | **Date last dose taken (c)** |
| 8. | | | | |  Complete  Incomplete  Unknown  N/A. | | --- | | | | |  |
| 9. | | | | | |  Complete  Incomplete  Unknown  N/A. | | --- | | | --- | --- | | | | |  |
| 10. | | | | | |  Complete  Incomplete  Unknown  N/A. | | --- | | | --- | --- | | | | |  |
| 11. | | | | | |  Complete  Incomplete  Unknown  N/A. | | --- | | | --- | --- | | | | |  |

| **symptom record**  (Rank on scale of 0-4: absent = 0; mild = 1; moderate = 2; severe = 3, life-threatening = 4, N/A = unable to assess) **EXTRA FORM USED; YES**  **NO**   | | | | | | | | | | | | | |
| --- | --- | --- | --- | --- | --- | --- | --- | --- | --- | --- | --- | --- | --- |
|  | **day 0** | **day 1** | **day 2** | **day 3** | **day 7** | **day 14** | **day 21** | **day 28** | **Day 35** | **day 42** | **day __** | **day __** | **day __** |
| **DATE** |  |  |  |  |  |  |  |  |  |  |  |  |  |
| 12. Fever in past 24h *(Y/N)* |  |  |  |  |  |  |  |  |  |  |  |  |  |
| 13. Weakness |  |  |  |  |  |  |  |  |  |  |  |  |  |
| 14. Muscle/joint aches* |  |  |  |  |  |  |  |  |  |  |  |  |  |
| 15. Headache* |  |  |  |  |  |  |  |  |  |  |  |  |  |
| 16. Anorexia |  |  |  |  |  |  |  |  |  |  |  |  |  |
| 17. Nausea* |  |  |  |  |  |  |  |  |  |  |  |  |  |
| 18. Vomiting |  |  |  |  |  |  |  |  |  |  |  |  |  |
| 19. Abdominal pain* |  |  |  |  |  |  |  |  |  |  |  |  |  |
| 20. Diarrhea |  |  |  |  |  |  |  |  |  |  |  |  |  |
| 21. Cough |  |  |  |  |  |  |  |  |  |  |  |  |  |
| 22. Pruritis |  |  |  |  |  |  |  |  |  |  |  |  |  |
| 23. Tinnutus* |  |  |  |  |  |  |  |  |  |  |  |  |  |
| 24. Behavioural changes |  |  |  |  |  |  |  |  |  |  |  |  |  |
| 25. “Flu” |  |  |  |  |  |  |  |  |  |  |  |  |  |
| 26. Other______________ |  |  |  |  |  |  |  |  |  |  |  |  |  |
| 27. Other______________ |  |  |  |  |  |  |  |  |  |  |  |  |  |
| 28. Adverse event  reported† *(Y/N)* |  |  |  |  |  |  |  |  |  |  |  |  |  |
| Initials |  |  |  |  |  |  |  |  |  |  |  |  |  |

****Only assess in children > 3 years of age. For children < 3 and those unable to answer, enter N/A.***

***† Adverse event reported if symptom is new or worsening and grade is > 2. Notify Kampala core facility immediately of all serious adverse events.***

| **UMSP clinical record form (2):** | | | |
| --- | --- | --- | --- |
| **Patient**  **Initials:** | **1. Study Number: U**|___|___|___|___|___| | **2. Day 0 Date:** |___|___|/|___|___|/|___|___|  ***day month year*** | **3. OPD Number:** ______________ |

| **physical exam record**  (Rank on scale of 0-4: normal = 0; mild abnormality = 1; moderate = 2; severe = 3, life-threatening = 4, N/A = unable to assess) | | | | | | | | | | | | | |
| --- | --- | --- | --- | --- | --- | --- | --- | --- | --- | --- | --- | --- | --- |
|  | **day 0** | **day 1** | **day 2** | **day 3** | **day 7** | **day 14** | **day 21** | **day 28** | **day 35** | **day 42** | **Day __** | **day __** | **day __** |
| **DATE** |  |  |  |  |  |  |  |  |  |  |  |  |  |
| 29. Temperature *(ºC)* |  |  |  |  |  |  |  |  |  |  |  |  |  |
| 30. Dehydration |  |  |  |  |  |  |  |  |  |  |  |  |  |
| 31. Jaundice |  |  |  |  |  |  |  |  |  |  |  |  |  |
| 32. Chest |  |  |  |  |  |  |  |  |  |  |  |  |  |
| 33. Abdomen |  |  |  |  |  |  |  |  |  |  |  |  |  |
| 34. Skin |  |  |  |  |  |  |  |  |  |  |  |  |  |
| 35. Tablet test |  |  |  |  |  |  |  |  |  |  |  |  |  |
| 36. Other__________ |  |  |  |  |  |  |  |  |  |  |  |  |  |
| 37. Other__________ |  |  |  |  |  |  |  |  |  |  |  |  |  |
| 38. Adverse event  reported† *(Y/N)* |  |  |  |  |  |  |  |  |  |  |  |  |  |
| ABNORMAL EXAM RECORD | | | | | | | | | | | | | |
| If abnormality noted on physical exam, describe all physical findings for the abnormal exam |  |  |  |  |  |  |  |  |  |  |  |  |  |
| **Initials** |  |  |  |  |  |  |  |  |  |  |  |  |  |

**** Follow age-based guidelines: Tablet test – > 9 mo; Heel-toe – > 2 years; Romberg – > 4 years. Answer N/A for younger children and uncooperative patients.***

***† Adverse event reported if exam sign is new or worsening and grade is > 2. Notify Kampala core facility immediately of all serious adverse events.***

| **UMSP clinical record form (3):** | | | |
| --- | --- | --- | --- |
| **Patient**  **Initials:** | **1. Study Number: U**|___|___|___|___|___| | **2. Day 0 Date:** |___|___|/|___|___|/|___|___|  ***day month year*** | **3. OPD Number:** _________________ |

| LABORATORY RECORD | | | | | | | | | | | | | |
| --- | --- | --- | --- | --- | --- | --- | --- | --- | --- | --- | --- | --- | --- |
|  | **day 0** | **day 1** | **day 2** | **day 3** | **day 7** | **day 14** | **day 21** | **day 28** | **day 35** | **day 42** | **day __** | **day __** | **day __** |
| **DATE** |  |  |  |  |  |  |  |  |  |  |  |  |  |
| 40. Parasite density  *(asexual parasites/ul)* |  |  |  |  |  |  |  |  |  |  |  |  |  |
| 41. Species |  |  |  |  |  |  |  |  |  |  |  |  |  |
| 42. Gametocyte density |  |  |  |  |  |  |  |  |  |  |  |  |  |
| 43. Haemoglobin*† *(g/dL)*  [grade] | [ ] |  |  |  |  |  |  |  |  | [ ] |  |  |  |
| Initials |  |  |  |  |  |  |  |  |  |  |  |  |  |

****(Grade on scale of 0-4: normal = 0; mild abnormality = 1; moderate = 2; severe = 3, life-threatening = 4)***

***† Any haemoglobin <*** 5g/dl measured after Day 0 is a serious AE. Notify Kampala core facility immediately of all serious adverse events.

| **RECORD OF ADDITIONAL MEDICATION GIVEN DURING STUDY** | | | | |
| --- | --- | --- | --- | --- |
| **Medication (a)** | **Indication (b)** | **Dose (c)** | **Duration (d)** | **Date started (e)** |
| **60.** |  |  |  |  |
| **61.** |  |  |  |  |
| **62.** |  |  |  |  |
| **63.** |  |  |  |  |
| **64.** |  |  |  |  |
| **65.** |  |  |  |  |
| 66. |  |  |  |  |
| **67.** |  |  |  |  |
| **68.** |  |  |  |  |

| **UMSP clinical record form (4):** | | | |
| --- | --- | --- | --- |
| **Patient**  **Initials:** | **1. Study Number:**  **U**|___|___|___|___|___| | **2. Day 0 Date:** |___|___|/|___|___|/|___|___|  ***day month year*** | **3. OPD Number:** __________________ |

| complete efficacy outcome | |
| --- | --- |
|  ETF     LCF   LPF   ACPR     N/A (Tick appropriate Incomplete Efficacy Outcome **)** | If failed, **STUDY DAY** of clinical failure (0-42) __________  **Reason for Clinical Failure:**   Severe malaria/danger signs/death with parasitemia Days 0-3 🡪 Specify criteria _________   Severe AE requiring change in treatment Days 0-2 excluding severe malaria/danger signs 🡪 Specify criteria _____________   Day 2 parasite count > Day 0 count   Parasitemia on Day 3 with temperature > 37. 5   Day 3 parasite count > 25% Day 0 count   Severe malaria/danger signs/death with parasitemia Days 4-42 🡪 Specify criteria ___________   Parasitemia on Days 4-42 with temperature > 37. 5 OR History of fever in past 24 hours.  Was patient admitted? Yes. __________ No. _______________  If yes specify date and diagnosis _______________________________ |

**Outcome Classification**

**ETF🡪** Assessed Days 0-3

**LCF🡪** Assessed Days 4-42 and previously not an ETF.

**LPF🡪** Assessed Day 42

and previously not an ETF

or LCF.

**ACPR🡪** Assessed Day 42 and previously not an ETF, or LCF.

| INCOMPLETE EFFICACY OUTCOME |
| --- |
|  **Excluded** - If yes, reason for exclusion: (Must tick one reason below) Last day of follow-up _____   Other antimalarial use: if yes, describe__________________________   Withdrew informed consent   Concomitant febrile illness: if yes, diagnosis_____________________   Death not due to malaria   Lost   Error/protocol violation made during follow-up that prevented outcome classification: ______________________________  Last day of formal assessment _______________________ |

Appendix 13. UMSP Adverse Record Form

| **adverse event record form** | | | |
| --- | --- | --- | --- |
| **Patient**  **Initials:** | **1. Study Number: U**|___|___|___||___||___| | **2. Day 0 Date:** |___|___|/|___|___|/|___|___|  ***day month year*** | **3. OPD Number:** ____________________ |

|  | ***Complete on day first reported*** | | | ***Complete on day first reported and update as needed*** | | | ***Complete on final day*** | | |
| --- | --- | --- | --- | --- | --- | --- | --- | --- | --- |
| **Event description (a)** | **Date of event onset (b)** | **Date event reported**  **(c)** | **Initials of person reporting** | **Maximum severity* (d)** | **Maximum relationship† (e)** | **Serious? ‡ *(Y/N)***  **(f)** | **Episodic?**  ***(Y/N)***  **(g)** | **Outcome †† (h)** | **Date event resolved‡‡ (i)** |
| **80.** |  |  |  |  |  |  |  |  |  |
| **81.** |  |  |  |  |  |  |  |  |  |
| **82.** |  |  |  |  |  |  |  |  |  |
| **83.** |  |  |  |  |  |  |  |  |  |
| **84.** |  |  |  |  |  |  |  |  |  |
| **85.** |  |  |  |  |  |  |  |  |  |
| **86.** |  |  |  |  |  |  |  |  |  |
| **87.** |  |  |  |  |  |  |  |  |  |
| **88.** |  |  |  |  |  |  |  |  |  |
| **89.** |  |  |  |  |  |  |  |  |  |
| **90.** |  |  |  |  |  |  |  |  |  |
| **91.** |  |  |  |  |  |  |  |  |  |
| **92.** |  |  |  |  |  |  |  |  |  |
| **93.** |  |  |  |  |  |  |  |  |  |
| **94.** |  |  |  |  |  |  |  |  |  |

*** d) Severity:** *Rank on scale of 1-4: mild = 1; moderate = 2; severe = 3, life-threatening = 4*

**† e) Relationship:** *Rank on scale of 0-4: none = 0; unlikely = 1; possible = 2; probable = 3; definite = 4*

**‡ f) Serious:** Criteria for serious AE: fatal, life-threatening, results in or prolongs hospitalization, results in significant or persistent disability or capacity requires medical / surgical intervention to prevent serious outcome. **If serious, report to Kampala core facility staff immediately. They will assist with patient management, assist with completion of serious AE forms, and report the AE if necessary.**

**††h) Outcome:** *Rank on scale of 1-5: resolved without sequelae = 1; resolved with sequelae = 2; AE still present at study end/discontinuation, but improving = 3; subject died = 4; unknown = 5*

**‡‡ i) Date event resolved:** *Complete on Day 28 – If AE still ongoing at end of follow-up, indicate in question (h).*
